# CellTool: An Open-Source Software Combining Bio-Image Analysis and Mathematical Modeling for the Study of DNA Repair Dynamics

**DOI:** 10.3390/ijms242316784

**Published:** 2023-11-26

**Authors:** Georgi Danovski, Teodora Dyankova-Danovska, Rumen Stamatov, Radoslav Aleksandrov, Petar-Bogomil Kanev, Stoyno Stoynov

**Affiliations:** Institute of Molecular Biology, Bulgarian Academy of Sciences, Acad. G. Bonchev Str. Bl. 21, 1113 Sofia, Bulgaria; tedy0890@abv.bg (T.D.-D.); rstamatov@gmail.com (R.S.); raleksandrov@bio21.bas.bg (R.A.); petarb.kanev@gmail.com (P.-B.K.)

**Keywords:** DNA repair, DNA damage response, live-cell imaging, laser micro-irradiation, FRAP, image analysis software, mathematical modeling, ALKBH2, PCNA, BARD1

## Abstract

Elucidating the dynamics of DNA repair proteins is essential to understanding the mechanisms that preserve genomic stability and prevent carcinogenesis. However, the measurement and modeling of protein dynamics at DNA lesions via currently available image analysis tools is cumbersome. Therefore, we developed CellTool—a stand-alone open-source software with a graphical user interface for the analysis of time-lapse microscopy images. It combines data management, image processing, mathematical modeling, and graphical presentation of data in a single package. Multiple image filters, segmentation, and particle tracking algorithms, combined with direct visualization of the obtained results, make CellTool an ideal application for the comprehensive analysis of DNA repair protein dynamics. This software enables the fitting of obtained kinetic data to predefined or custom mathematical models. Importantly, CellTool provides a platform for easy implementation of custom image analysis packages written in a variety of programing languages. Using CellTool, we demonstrate that the ALKB homolog 2 (ALKBH2) demethylase is excluded from DNA damage sites despite recruitment of its putative interaction partner proliferating cell nuclear antigen (PCNA). Further, CellTool facilitates the straightforward fluorescence recovery after photobleaching (FRAP) analysis of BRCA1 associated RING domain 1 (BARD1) exchange at complex DNA lesions. In summary, the software presented herein enables the time-efficient analysis of a wide range of time-lapse microscopy experiments through a user-friendly interface.

## 1. Introduction

Mammalian cells incur tens of thousands of DNA lesions on a daily basis [1,2]. To cope with this wide array of insults to genome integrity, cells have evolved an elaborate signaling network, termed the DNA damage response (DDR) and comprised of a several hundred proteins acting in multiple pathways [3,4,5,6]. The DDR integrates DNA repair and cell cycle control [4] to determine cell fate and, ideally, to prevent malignant transformation [1,2]. In general, distinct lesions activate dedicated repair machinery, that is, a particular set of repair proteins [1]. Notably, all repair reactions follow a common principle—repair factors are recruited to the site of damage, where they bind directly to DNA (e.g., PARP1 [7,8], Ku70/80 [9], RPA [10], etc.), or to other proteins in the vicinity of the lesion (e.g., ATM [11], ATR [12], 53BP1 [13], etc.), in order to perform their dedicated function for a given period of time before detachment [6,14,15]. It is therefore important to interrogate and understand the association and dissociation kinetics of any given repair factor, as these vary dramatically depending on the specific function of the protein, the nature of the lesion, and the cell cycle phase, among other aspects [1,4,16].

An informative approach to the study of DNA repair kinetics is through live-cell microscopy, particularly by fusing a protein of interest to a fluorescent reporter, such as green fluorescent protein (GFP) [17,18]. Upon the induction of DNA damage, the tagged repair protein would typically be recruited to the site of damage and form a bright focus, a process that can be visualized via time-lapse image acquisition under a fluorescence microscope. DNA damage can be introduced in multiple ways, which include, but are not limited to, CRISPR-Cas9- or restriction enzyme-mediated cleavage [19,20,21], ionizing radiation, ultraviolet (UV) laser micro-irradiation (micro-IR) [22], and treatment with chemical mutagens. Notably, cells also incur spontaneous DNA damage as a consequence of replication errors, transcription-replication conflicts, and byproducts of cellular metabolism, such as reactive oxygen species (ROS) and aldehydes [1].

As simple as such systems are in theory, there is no shortcut to obtaining meaningful results from time-lapse movies, which are often multidimensional (with spatial 3D and several channels over time) and fall under the term “big data”. A typical workflow for the analysis of such multi-gigabyte DNA repair experiments involves an arsenal of bioinformatics procedures. Initial pre-processing steps usually include denoising and bleaching correction. Thereafter, segmentation and tracking must be carried out at multiple levels: first at the level of cells, in order to “isolate” cells of interest from a larger field of view; then on the level of individual fluorescent foci, which represent DNA repair events. Obviously, segmentation and tracking at these different levels will require different parameters and sometimes even distinct algorithmic approaches. Although currently available software solutions, such as ImageJ/Fiji [23,24], ilastik [25], Baxter Algorithms [26], CellProfiler [27], and TrackMate [28], can accommodate such complex workflows, this requires various plugins to be utilized in separate steps. Since segmentation and tracking in live cells is inherently error-prone, it is essential that the processing results of all substeps be displayed simultaneously, which allows for on-the-fly error correction. After tracking the repair foci and obtaining the kinetics of the repair reaction, mathematical modeling of the process enables the quantification of various reaction parameters as well as their prediction. Through this approach, we previously measured and modeled the kinetics of over 70 DNA repair proteins at complex lesions [14,16]. By fitting mathematical equations to the observed kinetics and comparing them, we were able to obtain the sequence of recruitment and dissociation of DDR factors, providing a comprehensive temporal map, which revealed certain dependencies between repair processes and which could be utilized for studying the intricate molecular effects of cancer therapeutics.

The absence of a straightforward and freely available software specialized in the analysis of DNA repair kinetics data considerably delays insights into the matter. With this obstacle in mind, we developed CellTool, a software combining all the necessary tools for analyzing DDR kinetics derived from imaging experiments in a single package. We demonstrate two typical workflows for studying DNA repair with CellTool. In the first, we determined the previously unknown behavior of AlkB homolog 2 (ALKBH2), a demethylase involved in direct damage reversal, on UV laser-induced complex DNA lesions. We compared ALKBH2 kinetics to those of proliferating cell nuclear antigen (PCNA). In the latter workflow, we elucidated the exchange rate, rather than the recruitment and dissociation, of BRCA1 associated RING domain 1 (BARD1) at sites of complex DNA lesions. To this end, we employed fluorescence recovery after photobleaching (FRAP) [29,30], the analysis of which was greatly expedited by CellTool. While we describe the applications of CellTool in the context of microscopy-based DNA repair studies, the software and workflows presented herein can be employed for the analysis of diverse time-lapse imaging experiments, especially those exploring the kinetics of major cellular processes such as DNA replication and transcription, as well as dynamic phenomena such as phase separation.

## 2. Results

### 2.1. Interface and Functionality of CellTool

**Figure 1:**
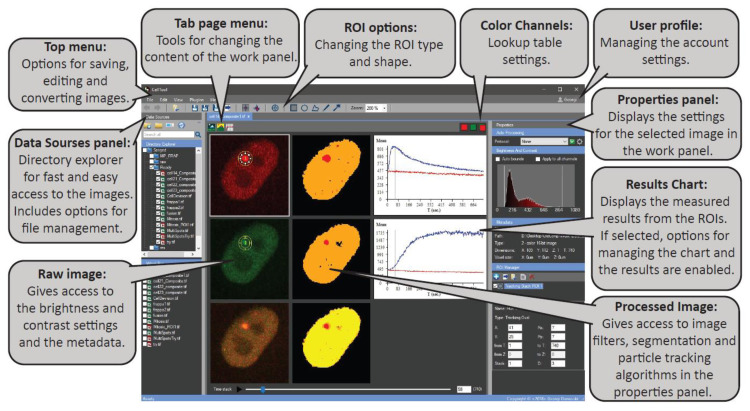
The main graphical user interface of CellTool.

We developed CellTool using the C# programming language. Its user-friendly graphical interface enables constant feedback between the different tasks to ensure fast and accurate image analysis (Figure 1). A generalized workflow in CellTool includes the following steps (for a full description, manual of use, and definitions, see Supplementary file S1).

(1) The user can add a work directory to the “Data Sources” panel. This integrated file manager is created for fast and easy access to any data supported by the Bio-formats library [31]. From there, an image or a group of images can be opened in a new tab in the central “work” panel.
(2) The image can be edited via different pre-processing steps, such as making substacks, projections, splitting, merging channels, or cropping.
(3) By clicking on the “Processed image”, different settings are enabled. Filters and binary operations can be applied, and the image can be segmented using global methods of segmentation and particle detection.
(4) Two types of regions of interest (ROIs), “Tracking” and “Static”, can be used to measure the objects. Different settings ROI settings can be applied, including a variety of shapes and a multilayer option.
(5) Results obtained from the ROIs can be immediately viewed on the “Results Chart” and can be exported as a tab-delimited text file. Here, a predefined formula can be used to eliminate some of the post-processing steps.
(6) Filtering, normalization, and visualization of the data from an image series can be performed using the “Results Extractor” plugin.
(7) Batch curve fitting of the acquired data to custom or predefined mathematical models can also be carried out using the “Results Extractor”.

The number and sizes of images that can be simultaneously opened in CellTool are not restricted by the software. Rather, these depend on the available RAM. We briefly compared the average time required to load an image file in CellTool and in ImageJ (version 1.54f). The same 0.8 Gb tif. file that had previously been saved in the respective software’s format took 1.5 s to open in CellTool versus 8 s in ImageJ. CellTool required 9 s to apply a Gaussian 5 × 5 blur compared to 3 s for ImageJ. We then loaded a 12 GB Imaris (ims.) file in both software tools. CellTool required 1:10 min to open it, compared to 3:42 min for ImageJ.

### 2.2. Analysis of PCNA Recruitment and ALKBH2 Exclusion from DNA Damage Sites via CellTool

To demonstrate the prowess of CellTool for the analysis of microscopy-based DNA repair experiments, we followed a previously established protocol for inducing DNA damage through the UV micro-IR of transgenic HeLa Kyoto cells [16]. Herein, we used a transgenic HeLa Kyoto cell line expressing PCNA-mCherry and ALKBH2-EGFP. We selected this pair of repair factors for a number of reasons. First, PCNA is a standard marker of DNA synthesis, including repair-associated synthesis, which produces unique localization patterns throughout different stages of the cell cycle [32,33]. Second, the kinetics of PCNA recruitment to DNA damage sites are well established, making this an appropriate reference for other repair factors [16]. Third, limited knowledge on ALKBH2 and the clinical relevance of direct reversal makes it an ideal candidate for further studies in the context of DNA repair [34,35]. Starting from a large field of view containing multiple cells in two channels, we eventually obtained the kinetics of recruitment and dissociation for both proteins, and then fitted mathematical equations describing these.

**Figure 2:**
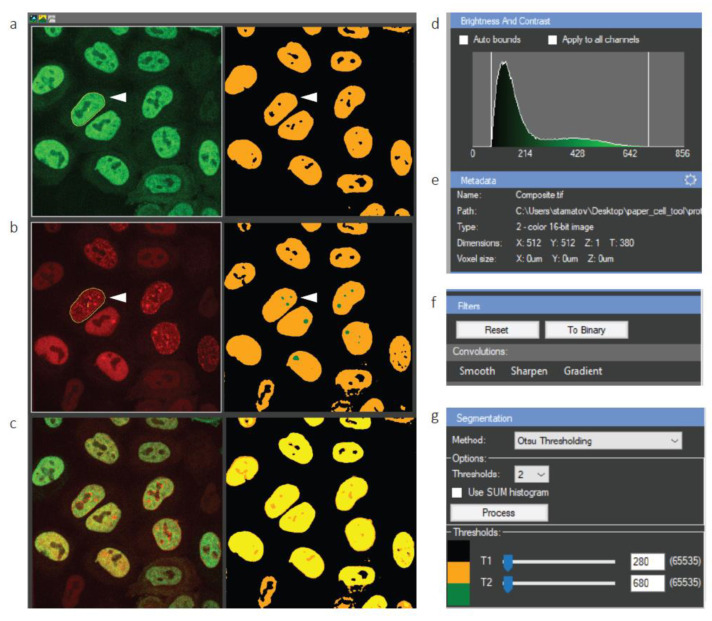
Tracking cell nuclei from a large field of view in CellTool. (**a**–**c**) Display of the raw data (**left**) and processed image (**right**). White arrowheads denote a cell of interest tracked over time; (**a**) GFP channel; (**b**) mCherry channel; (**c**) merged; (**d**) brightness and contrast settings of the raw image; (**e**) metadata, which can be edited (adjustment wheel in the top right corner); (**f**) image filters panel; (**g**) segmentation panel with a number of adjustable parameters.

Time-lapse micro-IR imaging experiments of the PCNA-mCherry/ALKBH2-EGFP cell line were visualized in CellTool, as shown in Figure 2a–c. The software enables us to simultaneously visualize the raw image next to the processed image (Figure 2a–c, right). The brightness and contrast can be adjusted at any time (Figure 2d), while the metadata display relevant information about the image file (Figure 2e).

The field of view contains 11 cells. In order to track a cell of interest and analyze it further, it must be segmented first. However, robust segmentation requires a smooth signal with as little noise as possible. To achieve this, CellTool users can choose from a collection of filters in the Filters submenu (Figure 2f). In this specific workflow, we used a Gaussian blur to remove the noise and aid in subsequent segmentation. The segmentation was carried out by Otsu thresholding, although KMeans segmentation can also be applied. Importantly, segmentation can be performed on multiple levels, with up to four user-defined thresholds. For example, the segmentation in Figure 2b-right utilizes two thresholds—one for cell nuclei (yellow) and another for PCNA foci (green).

Once segmentation is complete and the user is satisfied with the chosen thresholds, they can select an individual cell to track over time by choosing the magic wand ROI and simply clicking on the segmented cell (Figure 2a,b, white arrowheads). The chosen cell is automatically tracked, and its exact shape is delineated (Figure 2a,b, yellow lines). This cell can then be cropped and analyzed further.

**Figure 3:**
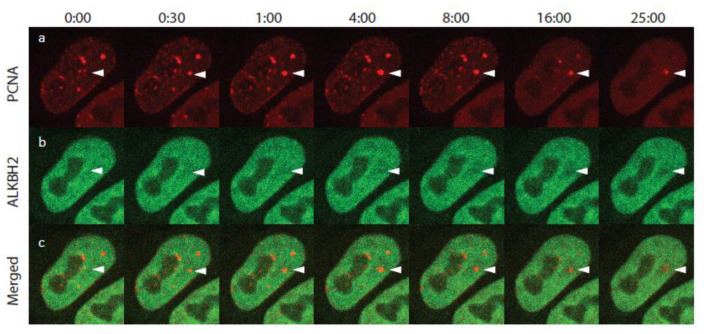
Selected frames from a time-lapse movie of ALKBH2 and PCNA after UV micro-irradiation. (**a**) PCNA-mCherry; (**b**) ALKBH2-EGFP; (**c**) merged. White arrowheads indicate the micro-IR site. The shown S-phase cell exhibited a number of PCNA replication foci. Following micro-IR, these foci dissolved due to checkpoint activation.

A time-lapse montage of the cropped cell is presented in Figure 3. White arrowheads indicate the area of micro-IR. Recruitment of PCNA-mCherry started within seconds of irradiation, reaching a maximum within 4 min, consistent with previous measurements [16]. The mCherry intensity then began to gradually decrease until the end of the imaging process. The behavior of ALKBH2 was strikingly unexpected, with depletion of the EGFP signal observed at the damaged site while PCNA was being recruited (Figure 3b, white arrowheads). To further investigate this peculiar dynamic, we analyzed each cell (tracked and cropped as described above) individually in CellTool, as follows.

**Figure 4:**
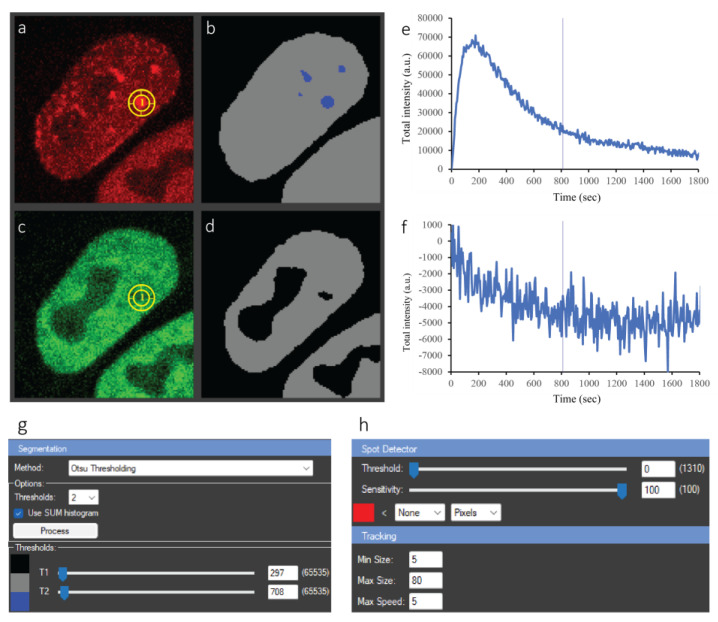
Analysis of ALKBH2 and PCNA dynamics after micro-irradiation of a single cell. (**a**) Raw PCNA-mCherry signal; yellow circle—tracked circular ROI of the damage focus; (**b**) segmentation result of (**a**) with two thresholds. Grey—whole nucleus, blue—foci. (**c**) Raw ALKBH2-EGFP signal; yellow circle—tracked ROI from the PCNA image, copied over to the same spatial coordinates on GFP. (**d**) Segmentation result of (**c**) with one threshold—depletion in the signal appears as holes. (**e**,**f**) Plots of the ROI intensity of the two channels over time. The straight line indicates the currently selected timepoint, to which the images in (**a**,**c**) correspond. (**g**) Segmentation panel; (**h**) tracking panel. All these items appear in a single window in CellTool, but are shown separately here for clarity.

First, Gaussian smoothing was performed to facilitate segmentation of the foci. Otsu thresholding was then applied, as is the case for whole cell tracking, but with the settings tailored to PCNA foci (Figure 4a,b). The blue dots in Figure 4b denote successfully segmented foci. The focus of interest is selected by clicking on the respective segmented dot. This simple step automatically triggers the tracking of the focus over time and places a tracking ROI on the corresponding coordinates of the raw image (yellow circle in Figure 4a). As stated above, PCNA and ALKBH2 exhibited contrasting kinetics—PCNA was recruited to the damage site, while ALKBH2 was depleted. The tracking of this depletion, which manifests as a decrease in fluorescence intensity at the site of micro-IR, presents a computational challenge. Using CellTool, we can handle the matter in two ways. First, simply clicking on the apparent hole in the segmented signal triggers the correct tracking, as is the case for a bright focus. Alternatively, if cells simultaneously express two (or more) fluorescently-tagged proteins of interest, which co-localize, as is the case for DNA repair proteins at the sites of complex DNA lesions, the selected tracking ROI(s) in one channel (e.g., PCNA-mCherry) can be transferred right away (by copy-pasting) to one or all other channels (e.g., ALKBH2-EGFP), eliminating the need for segmenting and tracking foci across all imaged channels individually.

Adding ROI(s) to the ROI manager triggers the plotting of data in the Results chart on the right side of the central panel (Figure 4e,f). Users can choose from a number of different plotting options in the Chart Properties panel. The *X* axis can be picked to represent the time in seconds, minutes, or hours, as well as the number of frames (T slices). The *Y* axis can be chosen to present changes in the minimum, maximum, or mean fluorescence intensity inside the selected ROI(s), as well as the variations in the area of the ROI(s) over the course of the time-lapse movies. Importantly, users can also define and implement custom formulas through the “Function editor” (accessed from the loop-shaped button next to the *Y*-axis options) for more complex imaging data analysis. To calculate the changes in total fluorescence intensity for PCNA and ALKBH2 (Figure 4e,f) in the workflow presented herein, we implemented a custom formula which compensates for photobleaching during both micro-IR and image acquisition (see Section 4.4). Importantly, the plots are dynamically linked to the raw and segmented data—any change in the processing, e.g., additional filtering, segmentation, or ROI adjustments, is automatically reflected in the plots.

Herein, we quantitatively analyzed the recruitment/depletion behavior of PCNA and ALKBH2. Figure 4e shows that PCNA was rapidly recruited at the beginning and reached a plateau, whereafter foci intensity began decreasing, which reflects dissociation from DNA damage sites. ALKBH2 depletion started immediately after UV irradiation, and the decrease in intensity stopped approximately 10 min later, with no recovery observed until the end of the experiment.

The segmentation and tracking settings for this experiment are presented in Figure 4g,h. All panels of Figure 4 are part of the same CellTool window, which simplifies the analysis. When the analysis is complete, the user can record the steps as a protocol and reuse it for other experiments with the same or different proteins of interest. Most importantly, the current work can be saved in the Tif file itself, and the next time the Tif is opened in CellTool, all processing will be loaded as well. This allows the user to continue working even after restarting the program.

**Figure 5:**
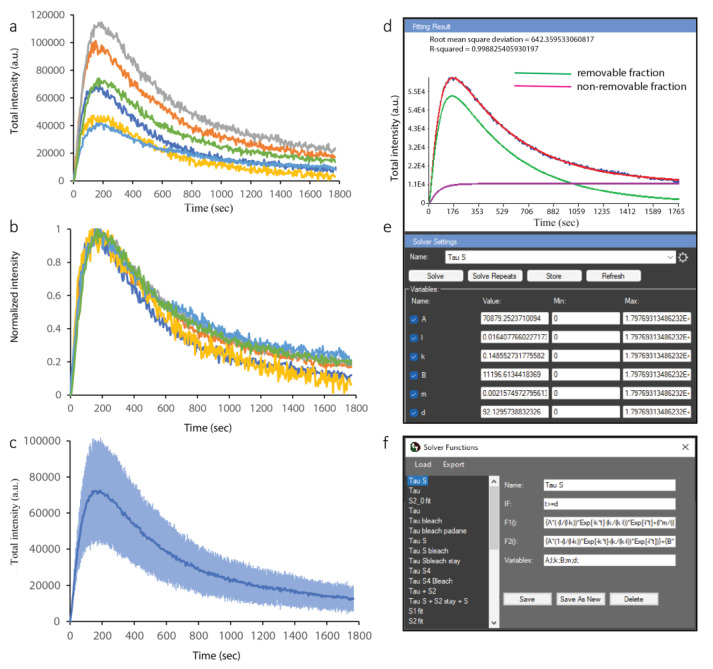
Analysis of PCNA data from multiple cells in the CellTool Results Extractor. (**a**) Original curves for PCNA recruitment in micro-irradiated cells. Curves with different colors are derived from different cells. (**b**) Normalized PCNA recruitment curves. (**c**) Averaged PCNA recruitment curve; the standard deviation is shown in a lighter shade of blue. (**d**) Fitting of the mathematical model (red) to the average curve (blue). The model is a combination of two components (green, purple). (**e**) The fitting interface, where initial values are chosen, and the fit are optimized automatically. (**f**) A pop-up window allows for the selection of a pre-existing model or the definition of a new one. All these items, except the pop-up window in (**f**), appear in a single window in CellTool, but are separated here for clarity.

We analyzed all micro-irradiated cells using the procedure described above and obtained the kinetics of PCNA and ALKBH2 for all of them. To compute a combined kinetics curve for the two proteins of interest, we took advantage of the Results Extractor in CellTool. This interface allows for simultaneous loading of the kinetics data from all cells located in a specified folder. By simply saving the data from each analysis in the Tif file, without further exporting or generation of intermediate files, the Results Extractor can access this information and load the kinetics curves in a single window. The results for PCNA recruitment are presented in Figure 5.

We first consider the range of signals from the different cells (Figure 5a). While the maximum level reached for each cell varied, the slopes of recruitment were similar between them. Therefore, obtaining a single, average kinetics curve for PCNA recruitment and the subsequent fitting of a mathematical model required normalizing each curve to the same range. Normalizing between 0 and 1 inside the Results Extractor is shown in Figure 5b, while the average curve with standard deviations for each time point is shown in Figure 5c. A notable similarity can be observed between the normalized curves, indicative of consistent and reproducible kinetics. Despite the intrinsic variation in cell shape, cell cycle stage, and the location of micro-IR, the responses of PCNA were strikingly similar (Figure 5b).

### 2.3. Fitting and Mathematical Modeling of PCNA Recruitment and ALKBH2 Exclusion from DNA Damage Sites via CellTool

CellTool allows for the straightforward fitting of simple or complex mathematical models to the acquired data. A previously defined and saved model can be selected from the drop-down menu of the fitting interface (Figure 5e), and initial parameters can be specified. The initial parameters are only needed to “nudge” the model optimizer in the right direction, and they may not be accurate at all. The solver will automatically optimize them and provide the best fit. The equation of the loaded model can be edited, or a new model can be defined in a pop-up window (Figure 5f and Supplementary file S1). We fitted a previously developed consecutive reaction chain (CRC) model [16] to the PCNA data. The CRC model can describe protein recruitment to damage as a series of up to three consecutive reactions, a period of residence at the lesion, and a reaction through which the protein is removed from the lesion. Importantly, this model can faithfully describe the kinetics of a factor that is recruited to the lesion through more than one mechanism, as we have previously shown. As is often the case, repair proteins may not dissociate completely from the damage site during the course of the imaging experiment. For example, 30 min time-lapse imaging is sufficient for obtaining the half-times of PCNA recruitment and removal from DNA damage sites, with a small focus still visible at the end. In such cases, the repair proteins kinetics are modeled as the sum of a removable and a non-removable fraction (Figure 5d). We provide a txt. file (Supplementary file S2) with a number of CRC model equations that can be load to the CellTool.

**Figure 6:**
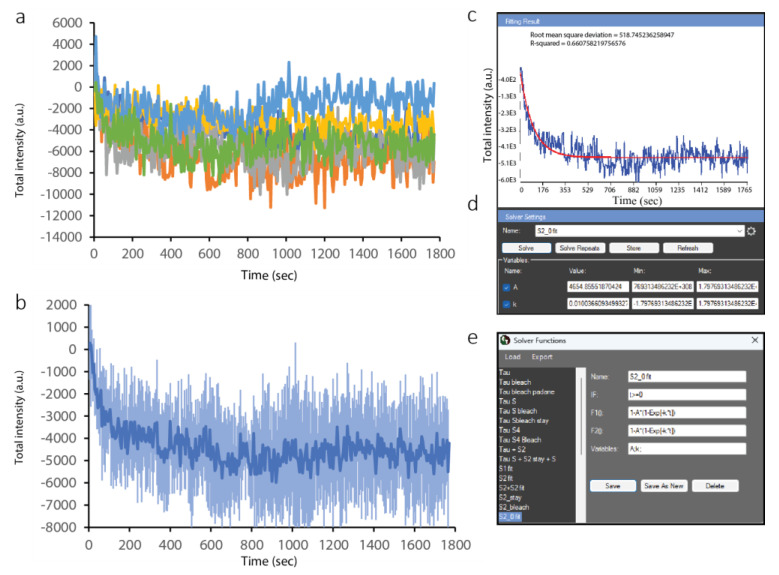
Analysis of ALKBH2 data from multiple cells in the CellTool Results Extractor. (**a**) Original curves for ALKBH2 depletion at the site of damage. Curves with different colors are derived from different cells. (**b**) Average ALKBH2 depletion curves. (**c**) Fitting of the mathematical model (red) to the average curve (blue); (**d**) fitting interface; (**e**) model selection interface.

To quantitatively analyze ALKBH2 dynamics at the sites of complex lesions, we repeated the processing steps described for PCNA in the Results Extractor. The individual curves (Figure 6a) show lower variation than those for PCNA, with the ensemble curve of depletion shown in Figure 6b. A much simpler, negative exponential decay model was used to fit the data (Figure 6c–e).

The depletion of ALKBH2 during PCNA recruitment was unexpected, in light of a previously suggested direct interaction between the two [36,37]. However, this interaction was shown to take place at DNA replication foci rather than during the repair of UV micro-irradiation-induced complex DNA lesions. The latter process requires elaborate coordination between different repair pathways, which may in turn necessitate the exclusion of certain factors from damage foci. The depletion of ALKBH2 from the site of damage suggests the existence of a mechanism that drives its extrusion, potentially in favor of another repair pathway.

### 2.4. Analysis of BARD1 FRAP Experiments Using CellTool

A comprehensive picture of repair dynamics is not limited to the recruitment and removal kinetics of repair factors. It has been shown that the exchange rates of different proteins on chromatin are of major relevance to genomic stability and even hold clinical relevance, as highlighted by the consequences of protein “trapping” at lesions and DNA–protein crosslinks, in general. The exchange rate of fluorescently tagged proteins can be determined via FRAP analysis.

To demonstrate the utility of CellTool for analyzing FRAP results, we determined the exchange rate of BRCA1 interaction partner BARD1 [38,39,40] at complex DNA lesions [41]. To this end, we employed a transgenic HeLa Kyoto cell line expressing EGFP-tagged BARD1. One hour after UV micro-irradiation, the formed BARD1 foci were photobleached, and their recovery was followed through time.

**Figure 7:**
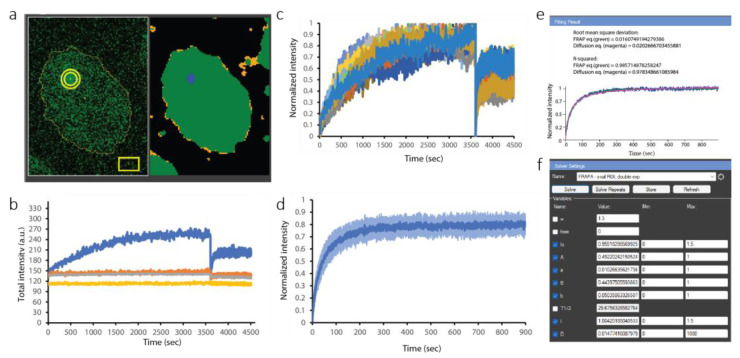
FRAP analysis in CellTool. (**a**) Left panel: original BARD1-EGFP signal. Yellow circle—ROI of the damage focus; yellow rectangle—ROI to measure the out-of-cell background. Thin yellow line—ROI of the cell shape. Right panel: segmentation of the image, with the damage focus in blue. (**b**) Representative plot of the ROIs in (**a**): total intensity within the ROI over the micro-irradiated region in blue; orange represents the intensity in the outer shell of this ROI; gray represents the ROI covering the whole nucleus; and yellow represents the rectangular ROI in the lower right corner (background). (**c**) Combined view of the BARD1-EGFP signal over time for all cells analyzed; the sudden drop in intensity indicates the moment of the FRAP. Curves with different colors are derived from different cells. (**d**) Isolated average recovery curve starting from the bleaching moment (right part of the curves in (**c**)). (**e**) Fitting of the mathematical model to the average recovery curve in (**c**), with the FRAP equation (green) and diffusion equation (magenta). (**f**) The fitting interface.

The results for a representative cell are shown in Figure 7a,b. Segmentation of the whole nucleus and focus was performed via two-threshold Otsu thresholding, as discussed for PCNA/ALKBH2 (Figure 7b). Three different ROIs were used for the analysis of this cell. The thin yellow line delineates the shape of the nucleus (BARD1 is a nuclear protein). This ROI enables us to measure the mean fluorescence (in arbitrary units based on intensity) of BARD1 inside the nucleus. The rectangular ROI is used for measuring the fluorescence background outside the cell. The actual focus is tracked by a different kind of ROI, called a stack ROI. It consists of two concentric circles (or more, if the user chooses). Such ROIs are especially useful for measuring FRAP results in order to discriminate between two protein populations: a fraction of proteins that bind to the damaged site and a freely diffusing fraction which does not bind. Both fractions contribute to the recovery of fluorescence intensity at the focus that is observed after photobleaching. By placing the inner circle exactly on the damaged site, it will contain the sum of the bound and mobile proteins, while the outer circle will contain only the diffusing, freely mobile proteins. Therefore, the difference in intensity between the inner and outer circle yields the fraction of bound protein.

Computationally, this is the difference between the blue curve and the orange curve in Figure 7b. The gray and yellow curves correspond to the EGFP signal inside the nucleus and the out-of-cell background, respectively. The lack of a decrease in the intensity measured for the whole nucleus over the 5000 s time-lapse acquisition imaging demonstrates that negligible bleaching, if any, occurred.

The inner and outer circle intensities were subtracted for each analyzed cell and combined in the Results Extractor (Figure 7c). The sudden drop at 3600 s was induced by photobleaching for FRAP analysis. We isolated only parts of the curves after bleaching, then normalized and averaged them (Figure 7d). Finally, we fitted a standard FRAP model (Figure 7e,f). CellTool allows for the fitting of FRAP results to pre-defined models, depending on the binding mode of the protein. The results can be fitted by single or double exponential models to derive the halftime of recovery or the exchange rate. In the case that the protein does not associate with damaged chromatin (which would be indicated by rapid restoration of the damage focus), the relevant equation would be the diffusion equation instead, which would yield a diffusion coefficient. When the protein does associate with the damage site, the diffusion equation provides an effective diffusion coefficient which reflects not only the diffusion, but also the duration of binding.

We fitted both the double exponent (FRAP equation) and the diffusion equation (green and magenta on Figure 7e,f, respectively). The exact equations are explained in detail in the user manual (theoretically derived in [42]). From the FRAP equation, we obtained the half-time of recovery, 25.68 s, and the mobile fraction, 0.955. The diffusion equation provided the effective diffusion coefficient of the mobile fraction: 0.0148 µm^2^/s. The half-time of recovery was much higher than what it should be for a completely mobile protein (which is typically on the order of 0.5–1 s). This 20–50-fold increase in the halftime of recovery confirmed the slow exchange rate of the bound fraction of BARD1 [41], which participates in DNA repair via homologous recombination.

## 3. Discussion

Time-lapse live-cell microscopy is an invaluable tool for the study of DNA repair and genomic stability, as the accumulation of various repair factors fused to fluorescent reporters can be followed through time and used as a read-out of damage and its resolution. However, the analysis of imaging data often represents a bottleneck for such experiments, emphasizing the lack of and need for a software that accommodates imaging files, incorporating all relevant processing and analysis steps, in a straightforward manner. We are confident that the development of CellTool addresses this major unmet need, providing a standardized approach to the analysis of micro-irradiation and FRAP, two well-established techniques for interrogation of the DDR via live-cell fluorescence microscopy.

The ease of analysis demonstrated in the two examples presented herein makes a case for CellTool as a top off-the-shelf software of choice for any researcher interested in the microscopy-based study of genomic stability. Functionalities beyond those employed in the analysis of PCNA/ALKBH2 and BARD1 are discussed in greater detail in the user manual. Our group previously employed CellTool to study the kinetics of over 70 repair proteins at micro-IR-induced complex DNA lesions [16]. The software allowed for convenient and rapid measurement of the formation and dissolution of repair foci. Through CRC mathematical modeling, which is also integrated into CellTool, we were then able to obtain halftimes of protein recruitment and removal, successfully comparing these between unperturbed conditions and under PARP inhibition. Further, modeling provided valuable insight into the mechanisms through which certain repair factors accumulate and dissociate from lesions.

An advantage of micro-irradiation is that it is generally performed at a pre-specified timepoint, allowing for precise temporal analysis of the ensuing repair processes. However, damage induction through other means, such as genotoxic therapy or DDR inhibitors, can also be studied using CellTool, as long as segmentable and trackable foci arise as a result of the damage or, more broadly speaking, a change in signal intensity ensues. In this regard, it should be mentioned that various repair factors form natural foci (in unperturbed conditions), which can also be followed through time. This is also the case for a myriad of replication- and transcription-related proteins, extending the application of CellTool beyond DNA repair. Importantly, CellTool is extendable—if a processing step is missing yet necessary, it can be written in the C# language, connected to the plug-in engine, and readily used. Currently, CellTool supports two major operating systems—Windows and Linux.

## 4. Materials and Methods

### 4.1. Cell Culture

HeLa Kyoto cell lines (RRID: CVCL_1922, sex: female) with stable expression of EGFP or mCherry N- or C-terminal-tagged proteins from BAC transgenes [43] were used. We studied two double-tagged cell lines: mCherry::PCNA + EGFP::ALKBH1 and mCherry::PCNA + EGFP::BARD1. The cells were cultured in Dulbecco’s Modified Eagle Medium (DMEM, Thermo Fisher Scientific, Waltham, MA, USA) with 10% fetal bovine serum (FBS) and 100 units/mL penicillin and 100 μg/mL streptomycin at 37 °C and 5% CO_2_. Before image acquisition, cells were transferred from culture flasks to MatTek glass-bottom dishes (MatTek Corporation, Ashland, MA, USA) at 20% confluence 48 h prior to experiments. The cells were then washed with PBS and supplemented with FluoroBrite DMEM medium (Thermo Fisher Scientific) with 10% FBS and 2 mM GlutaMAX (Thermo Fisher Scientific, Waltham, MA, USA).

### 4.2. UV Micro-Irradiation (IR) and Image Acquisition

We used the Andor Micropoint system (Oxford Instruments, Oxfordshire, UK) for IR experiments. It contains a 337 nm nitrogen laser which pumps a 365 nm dye laser. This provides 3.5 ns pulses with 150 μJ per pulse.

Ten pulses were used for IR, with 70% output attenuation of the 365 nm dye laser.

A constant temperature (37 °C) and CO_2_ (5%) were maintained during acquisition and IR.

Image acquisition was carried out on an Andor Revolution System (Oxford Instruments), with a Nikon Eclipse Ti-E inverted microscope (Nikon, Tokyo, Japan), a Nikon Perfect Focus System (PFS) (Nikon, Tokyo, Japan), a Nikon CFI Plan Apo VC 60× (NA 1.2) water immersion objective (Nikon, Tokyo, Japan), and an iXon897 EMCCD camera (Oxford Instruments, Oxfordshire, UK).

Three planes were acquired in Z, with 0.5 μm spacing and time-lapse intervals according to the application.

### 4.3. Fluorescence Recovery after Photobleaching (FRAP)

FRAP was performed as in [16], with the following settings: 60 μs dwell time, 6% of the maximum energy of the 488 nm laser (50 mW), 20 repeats. In brief, cells were first subjected to micro-IR, with images taken at specific time intervals until the fluorescence intensity peaked. The damage focus was then bleached, followed by rapid image acquisition to follow fluorescence recovery, in a single Z-plane.

### 4.4. Photobleaching Compensation

As extensively described in our previous work [16], we employed a circular ROI to measure the intensity of micro-IR-induced foci. Furthermore, this ROI had an outer “shell”, which covers the region immediately beyond the focus. The region that was bleached during micro-IR was greater than the region directly subjected to micro-IR, as exemplified by the same amount of photobleaching observed between the two regions at the time of micro-IR. By obtaining the difference in intensity between the main circle of our ROI (which outlines the focus) and the outer layer shell, we were, therefore, able to compensate for the bleaching that occurred both at the time of micro-IR and throughout time-lapse imaging.

### 4.5. CellTool Download and Code Availability

All relevant information regarding download, usage, license, tutorials, and source code are available at https://dnarepair.bas.bg/software/CellTool.

## Data Availability

The data generated and analyzed in this paper are available from the corresponding authors upon reasonable request.

## Supplementary Materials

The supporting information can be downloaded at: https://www.mdpi.com/article/10.3390/ijms242316784/s1. References [44,45,46,47] are cited in the supplementary materials.
