# Supplementary material for: CellTool: An Open-Source Software Combining Bio-Image Analysis and Mathematical Modeling for the Study of DNA Repair Dynamics
Source: Int J Mol Sci. 2023 Nov 26;24(23):16784. doi: 10.3390/ijms242316784 (PMC10706408; doi:10.3390/ijms242316784)
Supplement: Supplementary file 1 [file ijms-24-16784-s001.zip › Supplementary_S1.pdf]

CellTool

User Guide

Teodora Dyankova-Danovska and Georgi Danovski

TABLE OF CONTENTS

Introduction 4
    Supported image file types 4
    Output files 4
    License agreement 5
System requirements 5
Installation instructions 6
Updates 7
Profile settings 7
Top Menu 7
    File Menu 7
    Edit menu 8
    Help Menu 9
Data sources panel 10
Work Screen 12
ROI Manager 13
Raw image settings 17
    Brightness And Contrast 17
    Metadata 17
Processed image settings 18
    Histogram 18
    Convolution 18
    Segmentation 20
    Tracking Particles 21
    Spot Detector 21
Results chart settings 23
Auto Processing 24
Results Extractor Tab 25
    Results Extractor Solver 27
    Output file structure: 29
    Statistical equations used for the calculation of the results: 31
Examples 32
    Measuring DNA-repair foci 32
        Image analysis: 33

Further processing of the acquired data in the Results Extractor tab. 36
FRAP Analysis 38
Image analysis: 38
Further processing of the FRAPA data in the Results Extractor: 41
Developer guide 45
TifFileInfo class 46
ROI class 47
References: 48

INTRODUCTION

CellTool is a stand-alone open source software with Graphical User Interface that combines image analysis and real time data visualization. It has implemented algorithms for fast automatic segmentation of big time-lapse image stacks. This software is designed to process noisy images and is optimized for tracking objects such as cell nuclei and DNA repair foci over time. It has a plugin engine and an integrated file browser for fast and easy access to the images. The data obtained from the image analysis is dynamically displayed on a chart which allows the user to observe the results simultaneously with the image processing. Custom functions can be applied to the image processing which saves time and eliminates most of the post-processing steps of the measured data. The summarized results from the analysis of the image series can be graphically presented and fitted to a custom or predefined mathematical model. CellTool possesses intuitive graphical interface, implements multiple image processing algorithms and is capable of simultaneous data processing and data visualization greatly facilitating the efficiency of the image analysis. It is perfect for rapid and robust live cell image data analysis providing an all-in-one solution to numerous demanding tasks. That and the feature for simultaneous visualization of the results makes CellTool a very useful and user-friendly software for microscopy imaging facilities. For more information, visit our web site:

https://dnarepair.bas.bg/software/CellTool/

SUPPORTED IMAGE FILE TYPES

CellTool uses Bio-Formats library to read image data. It can open most of the available formats for microscopy images. CellTool supports 8-bit grayscale images and 16-bit grayscale images (RGB images are split into 3 separate grayscale images). List with the supported by Bio-Formats image file types is available at the following URL:

https://docs.openmicroscopy.org/bio-formats/5.8.2/supported-formats.html

OUTPUT FILES

CellTool uses 8-bit grayscale TIFF or 16-bit grayscale TIFF to store image data. The parameters obtained during the image analysis and the regions of interests (ROIs) from the ROI Manager are stored in the file metadata. The ROIs can be separately stored as ".RoiSet" file. The data calculated from the ROIs can be exported as TAB-delimited text file (with the button "measure" from the "ROI Manager")

	A	B	C	D	E	F	G	H
1	ImageN	T	T(sec.)	Z	Area1	Mean1	Min1	Max1
2	0	1	0	1	19536	80,26996	0	595
3	2	1	0	2	19536	110,8945	92	269
4	4	1	0	3	19536	148,6881	93	554
5	6	2	30	1	19536	79,66313	0	602

The first column is “ImageN” indicating the position of the current frame in the image stack. For each frame column “T” shows the time slice index, “T (sec)” – the time in seconds and “Z” – the position in the “Z stack”. Additionally 4 columns are added to the file for each ROI (ROI1, ROI2,…,ROIn): “Area” (the size of the ROI in pixels), “Mean” (the average intensity value in the ROI), “Max” (the maximal pixel intensity value) and “Min” (the minimal pixel intensity value).

	A	B	C	D
1	CTResults	D:\Data\cell14_Composite.tif		
2	Comments			
3	T (sec.)	Mean_ROI1	Mean_ROI2	Mean_ROI3
4	0	482.3107345	437.2729407	183.3303167
5	1	479.6836158	436.4929697	183.0361991
6	2	483.6271186	437.775245	180.4366516
7	3	480.1864407	437.0882917	180.2307692
8	4	488.9265537	439.0027572	178.9366516
9	5	479.6101695	431.3967507	179.6696833

Important: CellTool has the ability to export a selected portion of this data in separated TAB-delimited text file or even to process the data with custom mathematical functions and export the result with the “Export” button (Ctrl + E) in the tool strip menu. See Results chart settings.

LICENSE AGREEMENT

CellTool - software for bio-image analysis

Copyright (C) 2018, Georgi Danovski

This program is a free software: you can redistribute it and/or modify it under the terms of the GNU General Public License as published by the Free Software Foundation, either version 3 of the License, or (at your option) any later version.

This program is distributed in the hope that it will be useful, but WITHOUT ANY WARRANTY; without even the implied warranty of MERCHANTABILITY or FITNESS FOR A PARTICULAR PURPOSE. See the GNU General Public License for more details.

You should have received a copy of the GNU General Public License along with this program. If not, see http://www.gnu.org/licenses/ .

This software is using the following libraries:

LibTiff.Net | Bio-Formats | ikvm | OpenTK | NCalc| Math.NET Numerics|

Accord.Net| Microsoft Solver Foundation

SYSTEM REQUIREMENTS

Currently, CellTool can be installed on MS Windows 7 OS SP1 x64, MS Windows 8 OS x64, MS Windows 8.1 OS x64, MS Windows 10 OS x64. It requires Microsoft .NET Framework 4.5 or higher to be installed.

CellTool can be installed on other operating systems with Windows Virtual Machine.

INSTALLATION INSTRUCTIONS

1. Download and install “SetUp_CellTool.exe” from the following link:
   https://dnarepair.bas.bg/software/CellTool/CellTool/SetUp_CellTool.exe
   (Source code is available in GitHub)
2. Run the “CellTool.exe” shortcut from the desktop.
3. Login as "Admin" with password "123456".
   (We recommend that you change the admin password immediately)

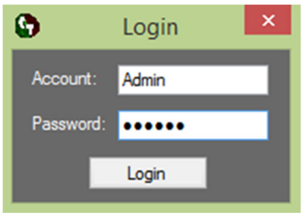

4. Create a new account by pressing the button “Create”. Change the admin password by pressing the button “Change Admin password”. To delete the existing accounts select them and press the button “Delete”.

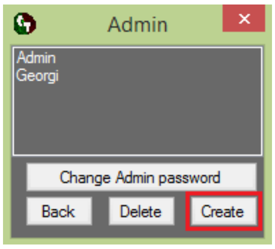

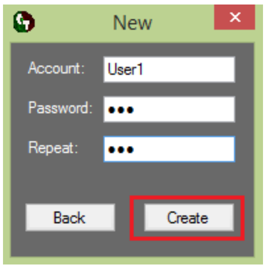

5. Return to the login screen by pressing the button “Back”.

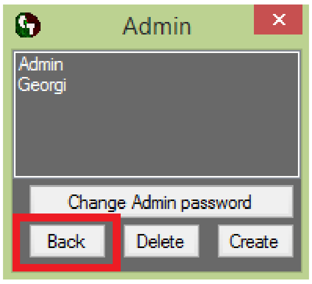

6. Login with the new account
7. Download a test image from the following link:
   https://dnarepair.bas.bg/software/CellTool/Program/test.tif

UPDATES

CellTool can be updated by accepting the update message when the software is started. It is recommended to keep the software up to date.

PROFILE SETTINGS

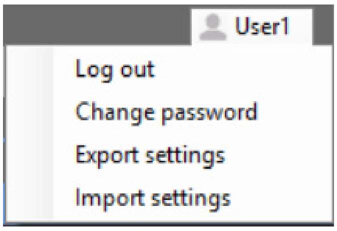

The user profile is located in the upper right corner of the program. For logging out and switching to another profile, “Log out” must be pressed. The password can be changed with the “Change password” option. **Important:** The account settings can be exported with “Export settings” as .CTProfile and the file can be transferred to another computer. The exported settings include the protocols for “Auto Processing”, “Hot Keys” and “Smart Buttons” (see Help menu) that are created by the user. Exported settings can be imported by pressing “Import settings” and browsing to the file.

TOP MENU

FILE MENU

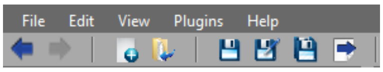

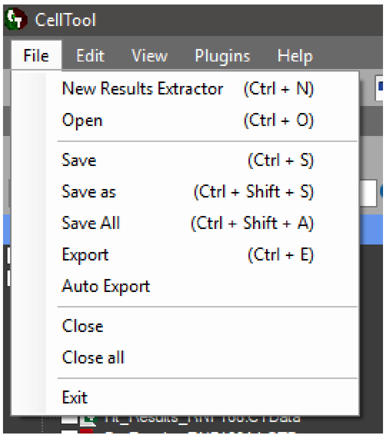

Images can be loaded to the program from the tool strip “Open” button (Ctrl + O) or from the “File” menu. From there the images can be saved or closed. The “Save” button saves the current active image, the “Save As” button saves the active image to a selected directory. **Important:** When attempting to “Save As” an image, the default is the directory of the image. The “Save all” button will save the changes made to all opened images. From the “File” menu the buttons “Close” and “Close All” will close the selected image or respectively all opened images. The button “Exit” can be used for exiting the application. When closing the program, it will exit without saving the unsaved images. The “Export” button can be used for exporting the measured results for the checked ROIs and will be discussed in the section “Results chart settings”. With the “New Results Extractor” button a special tab for post processing of the measured results is activated (see Results Extractor Tab).

With “Auto Export”, located in the “File” menu, the measured results will be automatically exported in the image’s directory for all color channels. Two Results text files will be saved per channel. First, the file that is usually extracted by pressing the “Export” button. It will carry the

name of the image and the color channel. It contains only the information from the settings chosen by the user (see Results chart settings). Second, the file that is exported when the "measure" button in the ROI Manager is pressed, it will carry the name of the image, the color channel and "_Results" at the end (see ROI Manager).

EDIT MENU

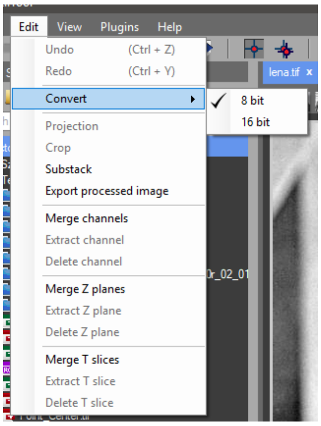

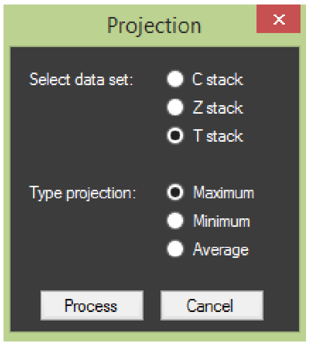

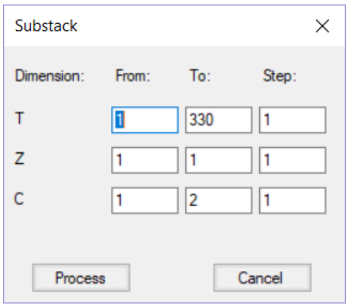

The "Edit" menu can be used to modify the image stack. From "Convert", the image type can be converted from 16 bit to 8 bit and from 8 bit to 16 bit. A selected ROI can be exported as a new image in a new tab by pressing the "Crop" button (see ROI Manager). Maximum Intensity, Minimum Intensity or Average Intensity projection can be performed. If "Z stack" is selected it will use the data from the Z stacks (all focal planes) for the calculation and the result image will be with 1 Z plane. For T (time) and C (color) stack projection the result image will be calculated analogically from the T or the C stack.

Two image stacks can be combined into one with the "merge" buttons. If "Merge Channels" is used the color channels of the two images will be stacked together. "Merge Z planes" will add the Z planes of the second image to the Z planes of the first image. "Merge T slices" will add the T planes of the second image to the T planes of the first image. With the "Extract channel/Z plane/T slice" and "Delete channel/Z plane/T slice" buttons the selected color channel, Z plane or T slice can be extracted to a new tab or removed from the image. The "Substack" button in the "Edit" menu can be used to create new image stack from the source image by determining the first and the final Z, T or C frame and the step for extraction (for example – every second T frame).

HELP MENU

Information about the license agreement of the software and the list with the used libraries is available in the “Help” menu. The button “Tutorials” opens new window with tutorials about the software.

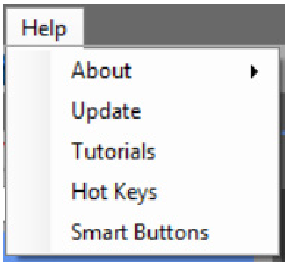

CellTool allows to manually select keyboard shortcuts. This option is available from the “Help”> “Hot Keys”. Every unassigned keyboard button in combination with the “Ctrl” key can be used. Shortcuts can be assigned to almost all tasks. Including the plugins and already existing auto processing protocols (one hot key per protocol) (see Auto Processing). Changes are applied when the “Save” button is pressed. If the key is already assigned a warning message will appear. “Smart buttons” can also be assigned to a specific CellTool command, plugin or auto processing protocol from the “Help”> “Smart Buttons”. By clicking on “Smart buttons” a new window will appear, from there they can be added, removed or rearranged with “Up” and “Down”. Once added the smart buttons will appear in the “Tool strip” menu after closing the window “Smart Buttons”.

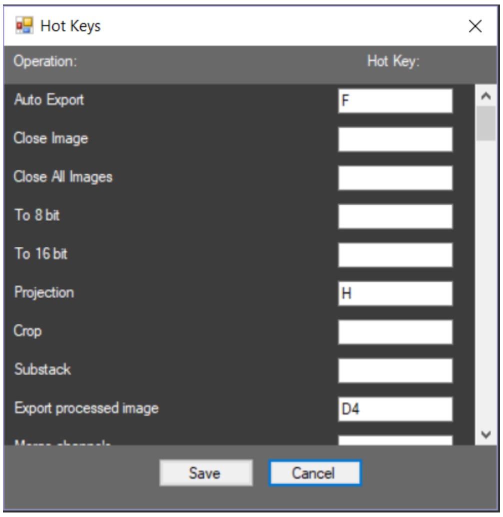

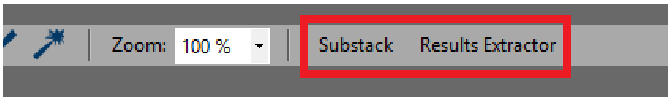

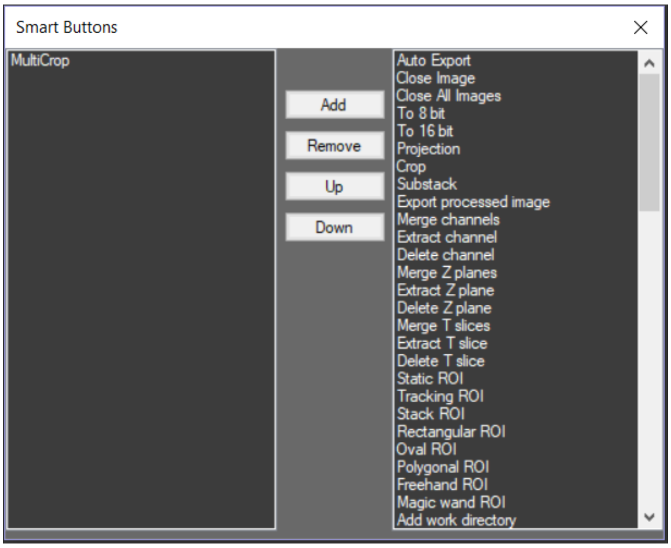

DATA SOURCES PANEL

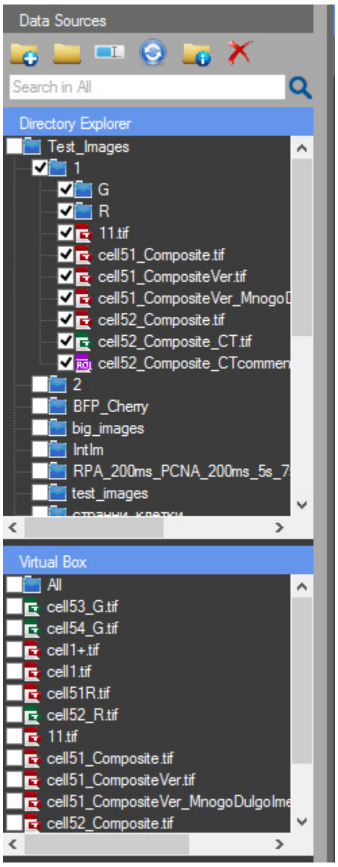

The "Directory Explorer" panel gives fast and easy access to the data. By pressing the "Add work directory" button a new directory can be added to the tree view. With right mouse click on an added work directory a context menu appears. From there multiple options are available. **Important:** An added work directory can be removed from the tree view with the "Release" button in the context menu and it can be completely deleted from its original directory with the "Delete" option.

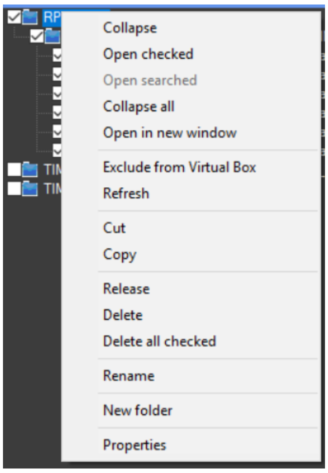

With right mouse click on an image or a subfolder in the added directory a slightly different context menu can be activated (There is no "Release" option there). With this controller, directories and files can be opened, copied, cut or pasted. They can be renamed or deleted. The properties of the image can also be viewed. **Important:** The options in the context menu differ, depending on if it's an image or a subfolder that is selected and if there are multiple images or subfolders checked.

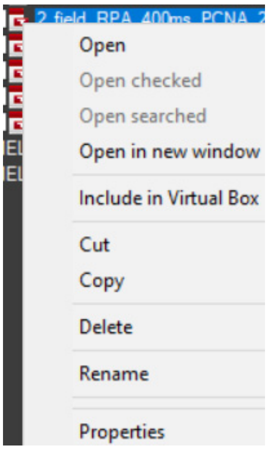

There are three types of Icons visible in the "Directory Explorer". The Red icon indicates a raw image. The green icon indicates a processed image. The red icon turns green if there is a text file with the same name as the raw image. This happens when the results from the chart (see Results Chart Settings) or from the ROI (see ROI Manager) are saved with the same name as the raw image. The purple icon indicates a ROI set created in the program. A ROI Set file can be opened in the active image by double click or "drag and drop". The RoiSet is channel specific (see ROI Manager).

A single image (raw or processed) can be opened with a double click on the name or icon or "drag and drop" on the work screen from the "Directory Explorer" of CellTool. A single

image can also be opened with "drag and drop" from the "File Explorer" of the computer to the work screen

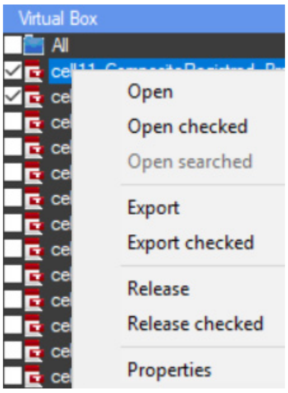

The "Virtual box" is the panel that allows for the extraction only of images and RoiSets from the selected work directory and its subdirectories. The images are automatically loaded to the virtual box by checking the folder they're in, from the directory explorer or by "drag and drop". The virtual box control has a context menu that can be accessed with a right mouse click. It can be used for opening images and releasing them from the virtual box. By pressing the "export" button the images can be copied to a new directory on the hard drive. If "Export" is clicked only the highlighted image can be exported to a new directory, chosen by the user. If "Export checked" is clicked all checked images can be exported.

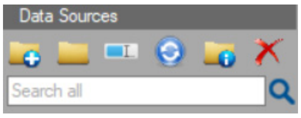

There can be a search for a specific string. The search can be performed in the "Directory Explorer" and/or the "Virtual Box", choosing between them can be done with a right mouse click in the search box.

Groups of images can be opened with the "Open" buttons in the context menu. When there are images in the tree view of the "Directory Explorer" or "Virtual Box" a context menu can be activated with a right mouse click on a single image or a group of checked or searched images. Then the images can be opened with "Open checked" or "Open searched". "Open in new window" can be used to open a folder from the tree view with the "File Explorer" on the computer. Dragging a folder from the "Directory Explorer" of CellTool to the work screen will open all the images in it.

WORK SCREEN

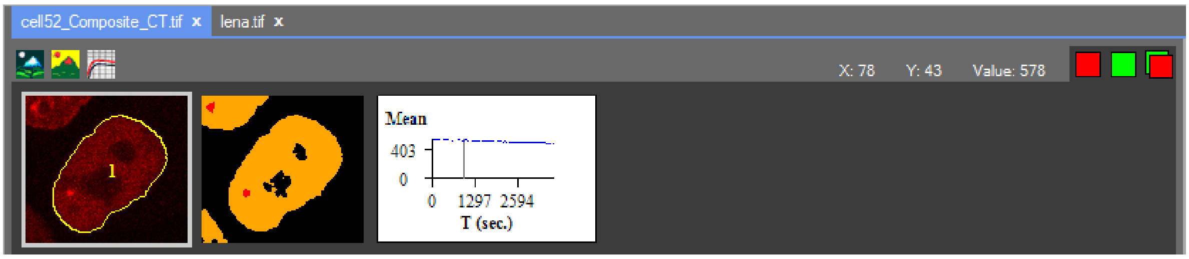

When the image is opened it is visualized in the central (work) panel. In the work panel multiple images can be opened in different tabs. The tabs can be rearranged by dragging. Additionally, an image can be closed by pressing the "X" button located next to the tab name.

In the work panel are the raw image, the processed image and the chart with the results. These three images are available for every color channel. All the channels of the image are displayed. The images can be resized by pressing the zoom in/zoom out buttons from the "View"

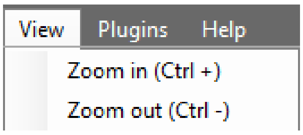

menu or with the hot keys "Ctrl + plus"/ "Ctrl + minus". This can be done with "Ctrl + mouse wheel up/down" or from the combo box in the tool 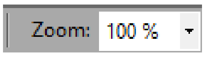 strip.

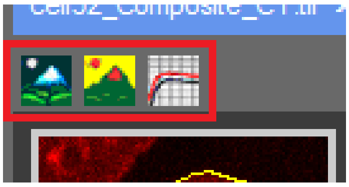

What is visualized on the work screen can be changed by pressing the 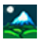 "Raw Image", the 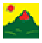 "Processed image" and the 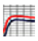 "Results chart" buttons in the tab page menu. If these icons are grey, they are not active (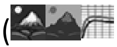) and the image or chart are hidden.

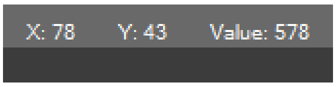

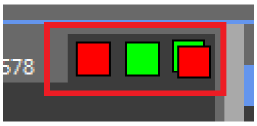

When pointing the mouse cursor at a certain pixel the coordinates and the value of the pixel are displayed at the end of the tab page menu. The "Lookup Table" can be changed with a right mouse click on the squares representing the color channels of the image. The color channels can be disabled with a left mouse click. They can be turned on at any time. **Important:** Even if one of the channels is not visualized any region of interest (ROI) added to the visible channel and then cropped (Edit menu) will be cropped from the whole image not only from the visible channel.

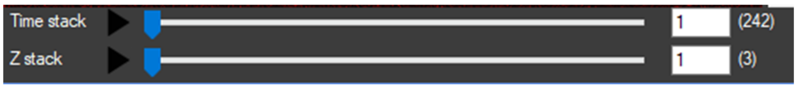

At the bottom of the work screen are two track bars. One indicating the current position in the "Time stack" and the other the current position in the "Z stack". The clip will start by pressing on the play button 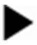, the icon changes to 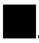. By clicking on the pause button 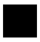 the clip pauses.

ROI MANAGER

The “ROI manager” is located in the “Properties” panel on the right edge.

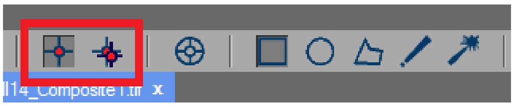

In the tool strip are two types of regions of interest (ROIs) – static and tracking. The static ROI can be enabled by pressing the “Static ROI” button. The static ROIs are located at the same coordinates in the time stack and the Z stack. They can be drawn only on the raw image. Every change made in the static ROI is applied for the whole image. The coordinates of the “Static” ROI can be adjusted for the whole image by dragging the ROI to another place or typing the desired coordinates in the “Options” panel. The “Options” panel activates by clicking on the number of the ROI in the image or on its name from the ROI manager. The tracking ROI can be enabled by pressing the “Tracking ROI” button. It can be used for measuring moving objects. Multiple static and tracking ROIs can be added. It is possible to switch between the two types of ROIs by clicking on the corresponding ROI.

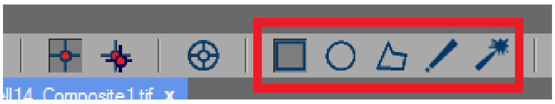

There are also different shapes for the ROIs: Rectangle, Oval, Polygon, Freehand selection and Magic wand. They can be found in the tool strip and can be used for measuring the intensity of the pixels inside them. All the different shapes are available for both type of ROIs.

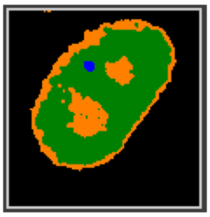

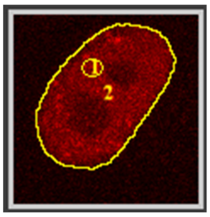

The “Tracking ROI” can be used only on the “Processed image”. When the image is segmented (see Processed Image Settings) every region in the “Processed image” is with different color. A mouse click on the object of interest ensures its tracking over time and will place a ROI that follows it. The ROI however will be placed on the “Raw image”. The coordinates of the “Tracking” ROI can be adjusted for a single frame by dragging the ROI in the correct place or typing the desired coordinates in the “Options” panel. If the shape of the ROI is “freehand” or “magic wand” it can be corrected on a single frame by manually redrawing it with the “freehand ROI” on the “Raw image” while pressing the keyboard button “Alt”.

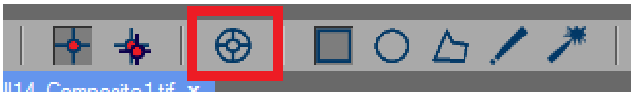

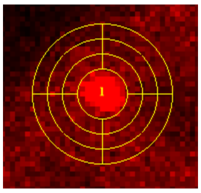

There’s also the “Stack ROI” option in the tool strip. This function allows for the creation of ROIs that are separated in layers. Each layer is measured independently. It can be applied by combining it with the “Tracking ROI” or the

“Static ROI”. The number of layers can be adjusted in the “Options” panel of the “ROI manager” (“Stack:”), the width in pixels of the layers can be adjusted from “D:”. Infinite number of layers can be added. The layered ROIs can be edited in the “Chart Series” panel when the “Results chart” is activated (see “Results chart settings”). Every layer is divided in four subROIs: “Left Up”, “Right Up”, “Left Down”, “Right Down”. Each of them can be included or excluded. **Important:** For the ROI to be visible in the “Chart Series” it must be added first (Ctrl + T).

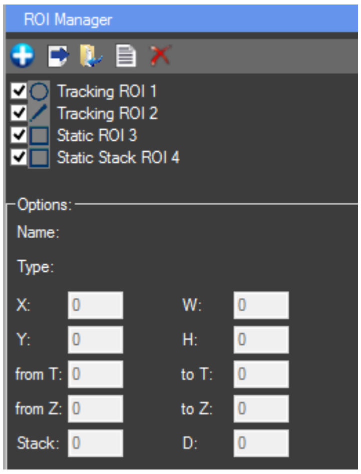

Once the ROI is created it can be added to the ROI list by pressing “Add new ROI” button or “Ctrl + T”. A not yet added ROI can be discarded with clicking the left mouse button somewhere in the ROI manager. For deleting added ROI/s is the button (Ctrl + D). Only selected (highlighted) ROIs can be deleted. In the “ROI Manager”, buttons for exporting the ROI set files and loading already existing ROI sets can be found. The ROIs are exported as .RoiSet files that contain the information for the ROIs coordinates in the image. Already existing ROI Set files can also be loaded to the active image from the “Directory explorer” of CellTool with double click or “drag and drop”. **Important:** The ROI set files are channel specific. Even if the same ROI set is used for all color channels, the exported RoiSet file will be for the channel of the image that was active at the time and if loaded will appear only on that channel.

The button “measure” (Ctrl + M) exports a TAB-delimited text file for the selected color channel that contains for every ROI the X axis results for T slice, T(sec), Z slice and for the Y axis, results for Area, Mean, Min, Max, see Output Files and Results Chart Settings. **Important:** This type of results file can’t be used for further processing in the “Results Extractor” tab.

Only checked ROIs will be exported or measured. **Important:** Only selected ROIs can be deleted Tracking Stack ROI 1. It doesn’t matter if they are checked or not. The ROIs are selected with left mouse click on the name of the ROI from the “ROI Manager”, not on the checkbox in front of the name. They can also be selected by clicking on the number of the ROI in the “Raw image”. ROIs can be multi selected with holding “Ctrl” and left clicking on the desired ROIs from the “ROI manager” or holding “Shift” and right clicking from the first desired ROI to the last, if all ROIs need to be selected.

In the “Options” panel of the “ROI Manager” additional information about the selected (highlighted, not checked) ROI can be found. From here the X and Y coordinates, width and height for the Oval and Rectangular ROIs can be adjusted. The frame from which the ROI will be enabled in the time/Z stack can also be specified from here. Measurements will be performed only for the

selected intervals. For the “Stack ROI”, the number of layers (“Stack”) and the width in pixels for the layers (“D”) can also be changed.

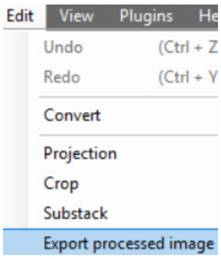

Important: The “ROI Manager” measures only the raw image. If measuring of the intensity in the processed image is needed, it has to be exported first. Pressing the “Export processed image” from the “Edit” menu will open it in new tab.

The “Crop” option in the “Edit” menu can be used for cropping ROIs out of the original image and exporting them as new images (for instance if there are multiple cells on the image). For this option to work the ROIs must be added to the ROI Manager. Multiple ROIs can be cropped at a time. **Important:** Only selected (highlighted) ROIs can be exported as new images 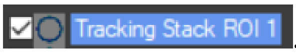

. It doesn’t matter if they are checked or not.

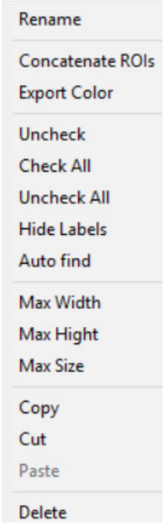

Context menus can be enabled: With right mouse click in the “ROI Manager” a generalized context menu for all ROIs will appear. With right click on a certain ROI in the “ROI Manager” a contexts menu containing specific options for that ROI will appear. From the context menus the selected ROI/s can be Copied, Cut and Pasted in other channel/image (ROIs can be copied and pasted between the two channels in the image or in images in other tabs). The “Rename” option can be used to add a comment/ label to the ROI. Also hot keys for “Copy” (Ctrl + C), “Cut” (Ctrl + X), “Paste” (Ctrl + V) and selecting all ROIs (Ctrl + A) can be used. **Important:** The order in which the ROIs are selected for “Copy” or “Cut” is the order they are going to be Pasted in.

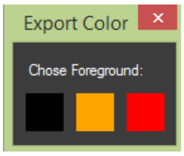

The button “Export Color” from the ROI Manager’s context menu can be used to measure only the pixels with specific color (chosen by the user) in the ROIs. The image must be segmented first (see Processed Image Settings). Results will be automatically saved in the image directory. Every ROI is exported as a new column.

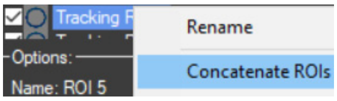

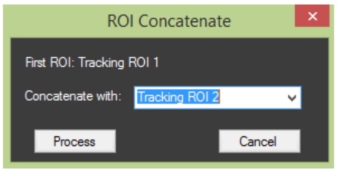

“Concatenate ROIs” algorithm (ROI Manager Context menu) can be used for merging several ROIs in one. **Important:** ROIs (“static” or “tracking”) can be added only to a “tracking” ROI. Also the time intervals must be properly adjusted before the concatenation (example: for the first ROI “from T: 1 to T: 10” and for the second ROI “from T: 11 to T: 300”). Only ROIs with the same shape can be merged.

In the ROI's context menu there are options for "Max Width", "Max Height", "Max Size" for a specific ROI. By choosing one of them, the program will automatically find and move the cursor to the slice from the image stack where the ROI is with its max width, max height, max size respectively.

"Static" ROIs can be added automatically to the ROI Manager by pressing the button "**Auto find**" (ROI Manager Context menu). The program takes the coordinates of the ROIs (FRAPA or microiradiation points) from the metadata of the image automatically if the images are acquired with IQ3 software (ANDOR). To import the ROIs from other type of software the user has to create a custom text file. The file must be named "RoiInfo.txt" and placed in the same folder as the image. It must contain the following information: <file name {with extension}>=<ROI shape {oval, rectangle, polygon, freehand}>(<coordinates>). The coordinates for oval and rectangular ROIs are the upper left X, the upper left Y, the width and the height values (Cell1.tif=oval(X, Y, Width, Height)). For polygonal and freehand ROIs the coordinates include the X and the Y values of every angle in the proper order (Cell2.tif=polygon(X1,Y1,X2,Y2,…,Xn,Yn)). ROI information can be stored in the same "RoiInfo.txt" file for more than one image.

For example:

Cell1.tif=oval(200,200,20,20)
Cell1.tif=rectangle(200,200,20,20)
Cell2.tif=polygon(250,250,250,280,280,280,280,250)
Cell3.tif=freehand(250,250,250,280,280,280,280,250)

RAW IMAGE SETTINGS

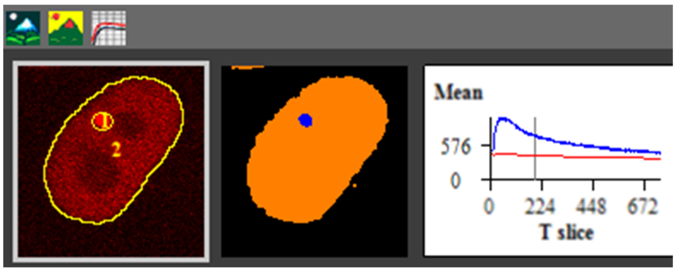

The raw image settings can be accessed with left mouse click on the raw image displayed in the work panel. The content of the “Properties” panel on the right will change and the “Brightness And Contrast” settings, the “Metadata” and the “ROI Manager” will become visible.

BRIGHTNESS AND CONTRAST

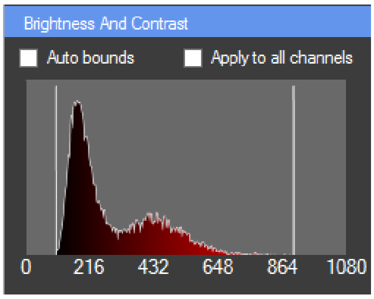

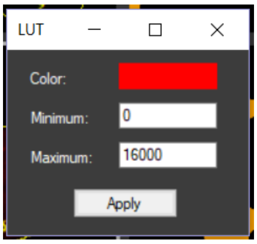

The brightness and contrast can be manually adjusted by dragging the two vertical white lines. With a right mouse click on the histogram a new pop up window “LUT” will appear. Here the lookup table of the channel can be changed. Also the settings can be adjusted by typing in the new values.

If “Auto bounds” is checked the brightness and contrast will be automatically adjusted for every frame. If “Apply to all channels” is checked adjusted settings will be applied to both channels.

METADATA

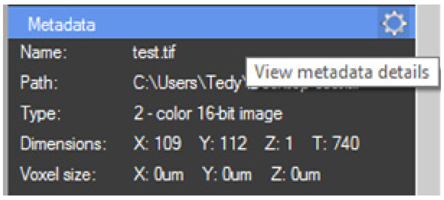

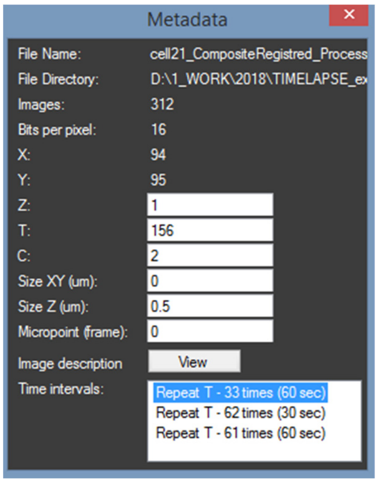

In the “Metadata” panel a short description of the image is presented. The directory of the image can be seen, its type and dimensions. If the “View metadata details” button is pressed a new window will appear with basic metadata of the image. Here the metadata can be edited. C, T and Z values can be changed and the time intervals in the image stack can be edited. **Important:** Sometimes, when the metadata tags are not correct, the Time stack (T) and Z stack (Z) values are switched. They can be adjusted here by typing them in the correct manner to switch them back. Pressing “View” next to “Image description” will open the full metadata of the image.

PROCESSED IMAGE SETTINGS

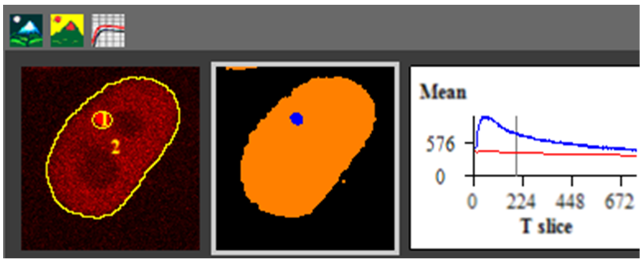

The processed image settings can be accessed with a left mouse click on the processed image displayed in the work panel. The content of the "Properties" panel will change and the image processing settings – "Histogram", filters ("Convolution"), "Segmentation", "Spot Detector" and "Tracking" particles will become visible.

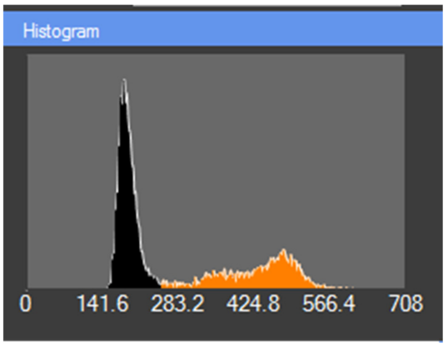

HISTOGRAM

This histogram is showing the distribution of pixels, based on their intensity, among the segmentation classes.

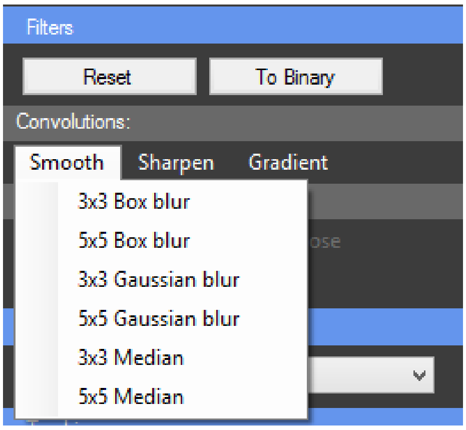

CONVOLUTION

Different image filters can be applied. They are divided in two major categories: Convolutions and Binary operations. The convolution filters multiply kernel matrix to the image and return the result image. Three types of convolutions are available – Smoothing, Sharpening and Gradient detection. In the following table all available kernel matrixes are shown.

Smooth

3x3 Box blur

1	1	1
1	1	1
1	1	1

5x5 Box blur

1	1	1	1	1
1	1	1	1	1
1	1	1	1	1
1	1	1	1	1
1	1	1	1	1

3x3 Gaussian blur

1	2	1
2	4	2
1	2	1

5x5 Gaussian blur

1	4	6	4	1
4	16	24	16	4
6	24	36	24	6
4	16	24	16	4
1	4	6	4	1

Sharpen

3x3 Sharpen

0	-1	0
-1	5	-1
0	-1	0

5x5 Sharpen

-1	-1	-1	-1	-1
-1	2	2	2	-1
-1	2	8	2	-1
-1	2	2	2	-1
-1	-1	-1	-1	-1

5x5 Unsharp masking

1	4	6	4	1
4	16	24	16	4
6	24	-476	24	6
4	16	24	16	4
1	4	6	4	1

Gradient

3x3 Edge detection

-1	-1	-1
-1	8	-1
-1	-1	-1

3x3 Gradient detection

-1	-1	-1
0	0	0
1	1	1

-1	0	1
-1	0	1
-1	0	1

3x3 Sobel operator

-1	-2	-1
0	0	0
1	2	1

-1	0	1
-2	0	2
-1	0	1

3x3 Embos

-1	-1	0
-1	0	1
0	1	1

0	1	1
-1	0	1
-1	-1	0

5x5 Embos

-1	-1	-1	-1	0
-1	-1	-1	0	1
-1	-1	0	1	1
-1	0	1	1	1
0	1	1	1	1

0	-1	-1	-1	-1
1	0	-1	-1	-1
1	1	0	-1	-1
1	1	1	0	-1
1	1	1	1	0

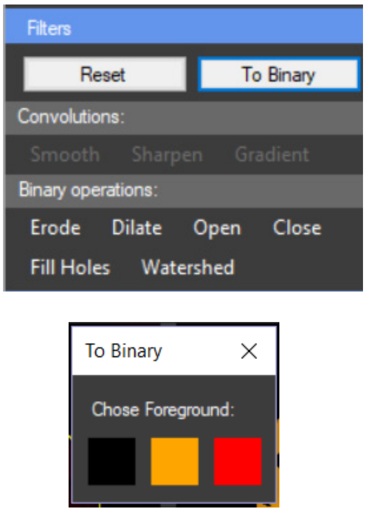

Binary operations can be applied after Segmentation only if the image is converted to binary. By clicking the "To Binary" button a new window will appear in which a foreground color has to be chosen. Afterwards the panel "Binary operations:" is activated from it different operations can be applied. "Erode" can be used for removing one-pixel layer from the foreground. "Dilate" can be used for adding one-pixel layer from the foreground. "Open" performs an erosion operation, followed by dilation. This smoothens the objects and removes isolated pixels. "Close" performs a dilation operation, followed by erosion. This smoothens the objects and fills in small holes. "Fill Holes" can be used to remove unwanted pixels with the intensity of the background from the object of interest. It can be combined with "Open".

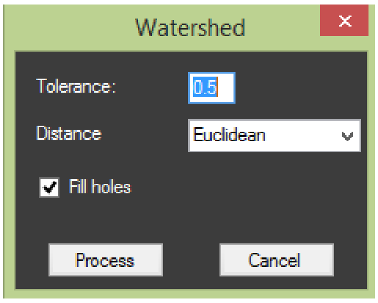

"Watershed" can be used on binary images in order to separate touching objects. Tolerance level can be changed to eliminate oversegmentation and undersegmentation. First part of the algorithm calculates distance map from the image. There are four types of distance transformation – Manhattan, Euclidean, Squared Euclidean and Chessboard. If "Fill holes" is checked that algorithm will be applied to the image before the "Watershed".

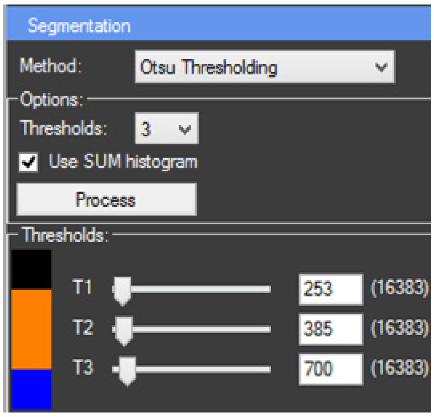

SEGMENTATION

From the "Methods" combo box a method for segmentation can be chosen. From the "Options" panel the number of desired thresholds can be selected. The algorithm will start when the "Process" button is pressed. If "Use SUM histogram" is NOT checked only the histogram of the current frame will be used for the calculations. Otherwise the histogram for the whole image stack will be calculated and then used for the segmentation. After the process of segmentation, the panel "Thresholds" will become visible. From there the calculated thresholds can be manually adjusted. The "Histogram" chart can be used to view the distribution of the pixels between the calculated thresholds.

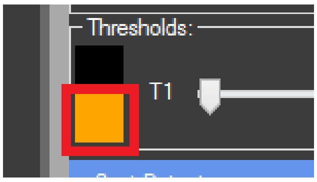

With a left mouse click on the colored button next to the threshold this class of pixels can be disabled and they will appear with their original color on the work screen. With a right mouse click on this button the color of this group of pixels can be changed.

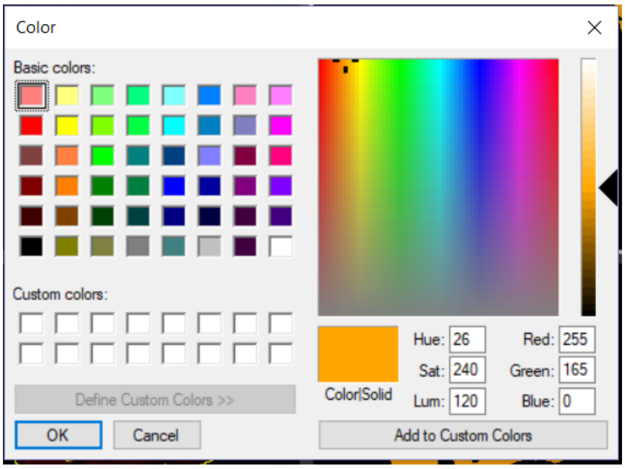

Important: If the same color is assigned to two or more classes of pixels they will be recognized as one class from the tracking algorithm.

TRACKING PARTICLES

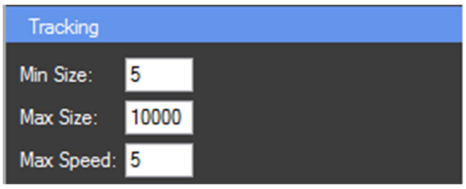

In the “Tracking” panel three parameters can be adjusted. That can help improve the accuracy of the tracking algorithm. “Min Size” is the minimum size in pixels of the tracked object. “Max Size” is its maximum size in pixels. If an object with the desired size is not found on some of the slices in the image, the last calculated ROI will be used there. The algorithm is calculating the center of mass for each T slice where the object appears. The object is added to the tracking ROI only if the distance of translation in pixels is less than the “Max Speed” parameter. If the object of interest is moving fast, the speed should be increased. The tracking algorithm starts from the selected T slice and moves forward and backward to the end or beginning respectively of the image stack.

SPOT DETECTOR

The “Spot Detector” panel can be activated by clicking on the processed image. The spot detector can be used for detection of brighter or darker spots in the image. It analyses the histogram of the image and calculates the zones of interest. Then with the other features of CellTool the detected regions can be tracked and measured overtime or just traced and measured, depending on the type of the image (single image or stack).

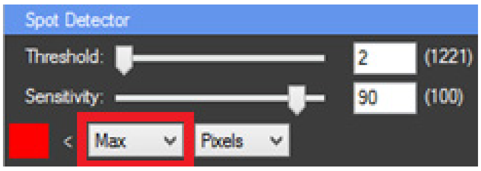

The first step in using the “Spot Detector” is to set a “starting point” for the algorithm which can be Min (0 intensity), Max (the highest intensity value that occurs in the image) or a threshold (T1 for the first threshold, T2 for second…, T4 for fourth).

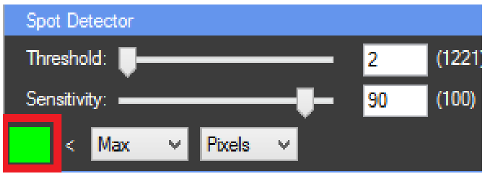

The color of the detected spots can be changed by right mouse click on the “color button”.

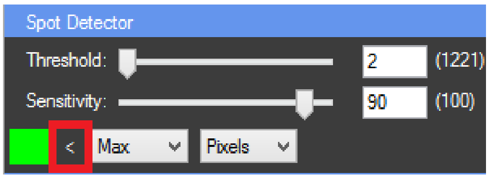

The next step is to choose the zone of interest with higher intensity (“>”) or with lower intensity (“<”) then the selected starting point. Switching between higher (“>”) or lower intensity (“<”) can be done by clicking on the 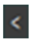 button.

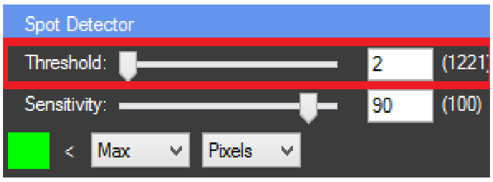

The algorithm starts from the “starting point” and counts the pixels. If the number of pixels is less than the selected threshold value, the algorithm checks the next intensity pixel group (with lower or higher intensity depending on the selected option – “<” or “>”). This process continues until it reaches an intensity pixel group with number of pixels higher than the selected threshold value. All checked pixels are included in the zone of interest.

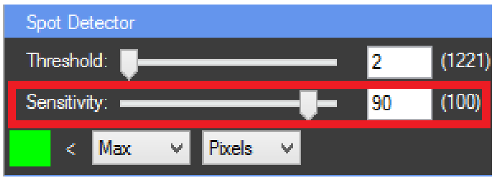

The histogram curve can be smoothed by lowering the sensitivity value. This will reduce the number of pixel intensity groups by merging the closest groups into one depending on the selected sensitivity value. This will affect the quality of the spot detection process

RESULTS CHART SETTINGS

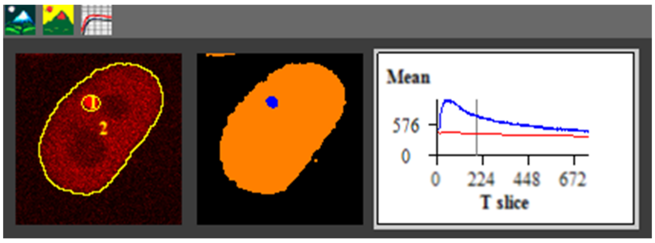

The results chart settings can be accessed by a left mouse click on the results chart displayed in the work panel. The content of the properties panel will change and the chart settings will become visible.

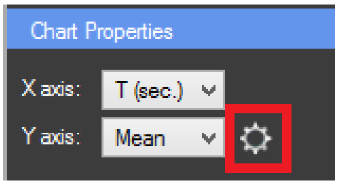

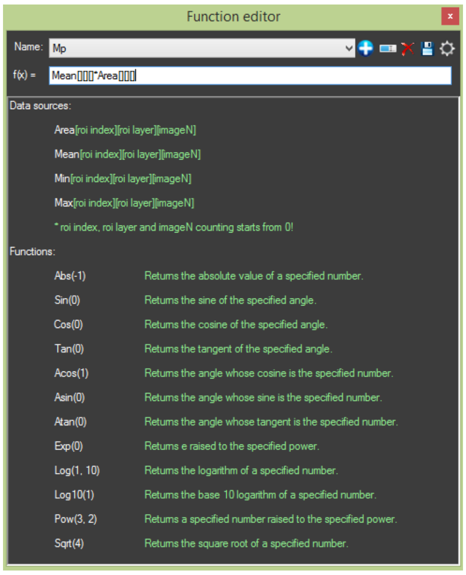

What is visualized on the axis can be changed. For the "X axis" it can be chosen between T slice, T (sec.), Z slice, T(min.) and T(hr.). To use T (sec.), T (min.) and T (hr.), the time steps (if there are any) need to be set in the metadata. For the "Y axis" it can be chosen between Area, Mean, Min, Max, Total or a custom function. If "Area" is chosen on the chart the size of the ROIs will be displayed. "Mean" is average pixel intensity value, "Min" and "Max" are respectively the minimal and maximal intensity value of the pixels in the ROIs. "Total" represents the "Mean" value multiplied by the "Area" of the ROI. Custom functions can be applied by writing their formula in the "Function editor".

The **function editor** window can be accessed by pressing the "Function editor" button next to the Y axis combo box in the "Chart Properties" panel. The software will calculate the selected function for each ROI and will display it on the chart. The function editor can be used to "Add new function". When the is pressed a pop up window appears and in it the name of the new function can be typed. Existing functions can be edited, after any changes are made they can be saved by pressing the "Save function" (**Important:** if saving an edited function as a new one is needed the "Add new function" button must be pressed, the name can be changed and the function will be saved as a new one). The functions can also be renamed or deleted. In the text box "f(x)", a custom function can be typed by using the CellTool format described in the software.

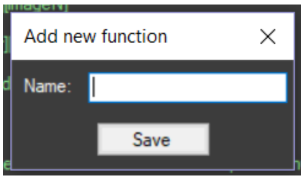

Functions Mean, Max, Min and Area must be written with "[ROI index][ROI layer][ImageN]" in the end. If the function has to be calculated for all ROIs and frames, empty

brackets are needed: Mean[][][] * Area[][][]. If constant ROI index, ROI layer or frame is needed it has to be specified. **Example**: If the first ROI needs to be subtracted from the mean value of every ROI the following function is used: Mean[][][] – Mean[0][][]. This rule can be applied also for the layer and the frame. The function can be checked for errors with the "Check for errors" button. All functions available in the Ncalc library can be used.

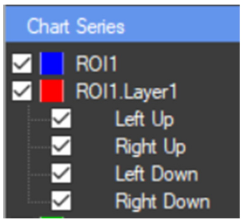

The color of the chart series can be changed by a right mouse click on its name in the "Chart Series" panel. Also the series can be enabled or disabled by checking or unchecking them.

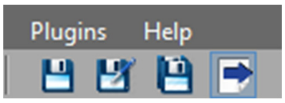

The "Export" button (Ctrl + E) can be used to **save the data visualized on the chart** as a TAB delimited text file. By pressing it a safe file dialog appears and in it the user can choose a specific name. The file contains the information from the settings chosen by the user. For instance, for the X axis only Tsec. and for the X axis only Mean. This file can later be used by the "Results Extractor" tab.

AUTO PROCESSING

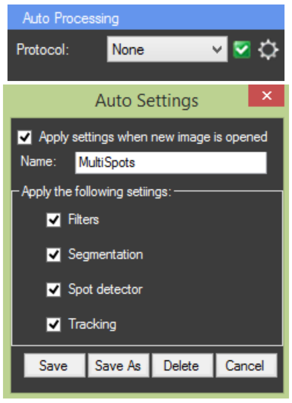

At the top of the properties panel is a locked panel for "Auto Processing". It can be used for storing settings. After image processing the steps of the process can be saved by creating a protocol. This can be very helpful for fast processing of similar images. A protocol is created by clicking on the next to "Protocol" in the "Auto Processing" panel. Then a pop up window "Auto Settings" appears. There is a text box to type the name of the protocol and an option to choose which settings will be saved in the profile by checking or unchecking them. A combination of filters and segmentation methods for instance. If the "Apply settings when new image is opened" is checked, the settings will be automatically applied to every newly opened image. The protocol is added by clicking "Save". After creating the first protocol, to create another one, the name of the protocol must be changed and "Save As" must be clicked. If only the "Save" button is clicked the new protocol will be written over the old one. Numerous profiles can be saved. A protocol can be deleted by pressing the "Delete" button.

RESULTS EXTRACTOR TAB

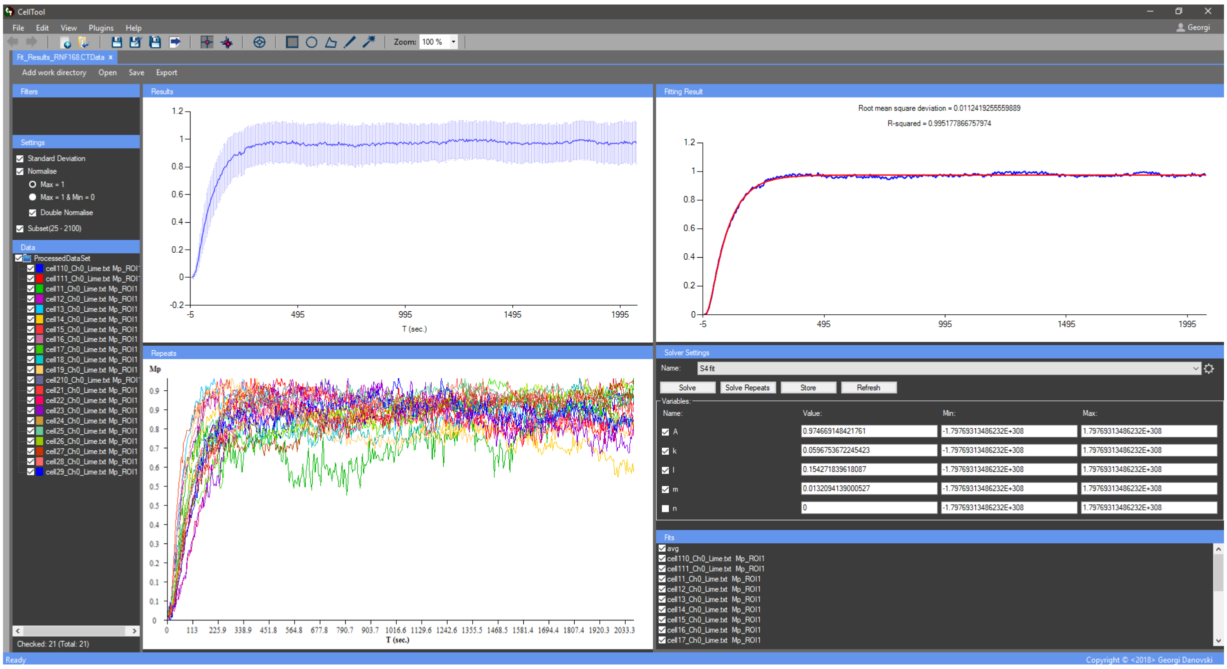

This feature can be accessed from the main menu by pressing the button “File>New Results Extractor” or from the “New Results Extractor” button in the tool strip. It can be used for data processing of the results from the image analysis. It uses as **source data** the text files acquired during the image analysis with CellTool (the text files containing the exported information from the “Results chart” in CellTool, the ones generated by pressing the “Export” button (Ctrl + E) in the main menu or “Auto Export” from the “File” menu in CellTool.)

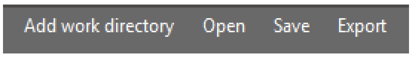

The results text files can be loaded to the “Result Extractor” with the “Add work directory” button in the menu of the tab. By pressing that button a directory browser appears and from it the user can browse to the folder with the processed images and their text files. **Important:** If the last processed image is still open in CellTool, the default folder in the directory browser is that of the processed image. By clicking “OK” the information of all the processed images in this folder will be loaded in the tab automatically. The data from these files will be displayed on the “Repeats” chart. There can be multiple folders from different directories added with the “Add work directory” button. This can be helpful for combining data from experiments from different days for instance. Once loaded to the tab the data can be saved with “Save” as a text file with “.CTData” extension. All the settings and changes will be stored and can be viewed later in CellTool. The saved “.CTData” files can be reloaded by pressing the “Open” button. The results from the tab can be exported with “Export” as a TAB-delimited **output text file** and used in another software if needed. The information for the calculated fits, if there is any, and the formulas used for fitting are also exported with

everything else in the TAB-delimited text file. **Note:** The "Open", "Save" and "Export" functions can also be activated from the main file menu or the tool strip of the program.

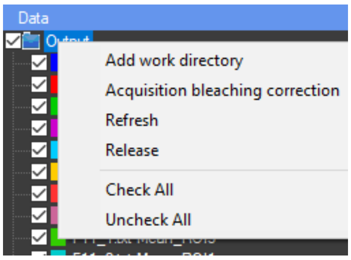

With a left mouse click in the "Data" panel in the tab a context menu appears. Files can also be loaded to the tab by pressing the "Add work directory" button from there and browsing to the directory where the files are located. The "Refresh" button reloads the text files in the tab. It can be used for loading text files from images from the same data set processed after the tab is opened. The "Release" button realeses folders from the panel. There is also the "Acquisition bleaching correction" option for **normalizing FRAPA data** by applying two formulas, one for **Double Normalization** and one for **Full Scale Normalization** [2] (See Further processing of FRAP data). Three parameters need to be predefined

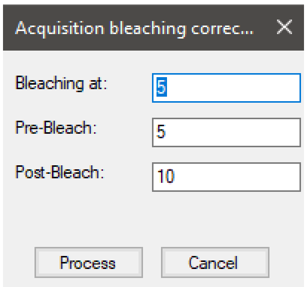

in the "Acquisition bleaching correction" window. In "Bleaching at:", the number of the frame of the bleaching. In "Pre-Bleach:" the amount of frames before bleaching. There must be at least one frame before the bleaching. In "Post-Bleach:" the amount of frames after bleaching.

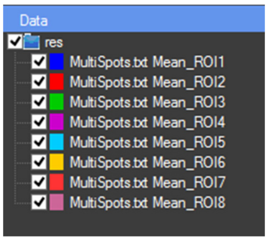

CellTool will calculate automatically the average curve and the standard deviation. They will be displayed on the "Results" chart in the tab. A certain sample curve from the "Repeats" chart can be eliminated from the average curve("Results" chart) by unchecking its data series in the "Data" panel. A certain curve can be highlighted (Bolded) by clicking on it in the "Repeats" chart or selecting its name in the "Data" panel. The Y values of both charts can be "Area", "Mean", "Min", "Max", "Total" or a custom function, depending on the chosen option during the image processing stage. **Important:** Theese parametars can't be changed in the "Results Extractor" once they are saved during the processing of the images.

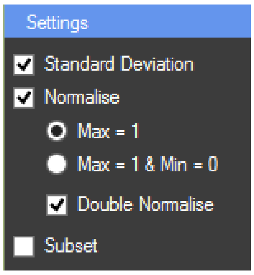

The normalised results can be viewed by checking the checkbox "Normalise" in the "Settings" panel. If "Max = 1" is checked, the values will be calculated by dividing the Y value of each data point by the maximal Y value of the sample. If "Max=1 & Min=0" is checked, additionally the minimal Y value of the sample (on the "Repeats" chart) will be subtracted from each data point of the Y value, followed by dividing the maximal Y value. If activated, the "Double Normalise" option will recalculate the

average curve (on the "Results" chart) in order to normalise it by dividing the Y value of each data point by the maximum Y value.

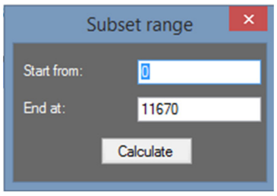

A subset can be created from the data set depending on the X values (T slice, T(sec.), Z slice, T (min.) or T(hr.)) by using the "Subset" option. When the checkbox next to "Subset" is checked, a popup window "Subset range" appears. In this window the data set can be shortened. The original data set can be restored by unchecking the checkbox.

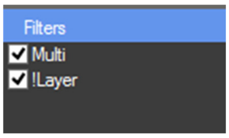

Filters can be used to sort the "Data" tree view. They can be added with a right mouse click in the "Filters" panel. Only the series that contain the text written in the filter (in the name or in the comment) will be active. Series that contain a specific text can be eliminated by adding "!" in the beginning of the filter text.

RESULTS EXTRACTOR SOLVER

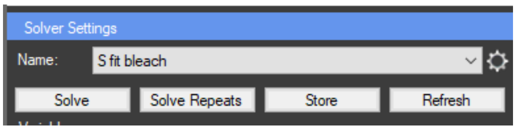

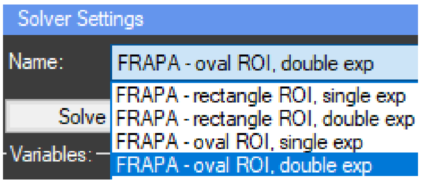

The "Results extractor" can be used for fitting the results to a predefined or custom mathematical model. The predefined mathematical models are embedded in the "Results Extractor". There are four predefined mathematical models for fluorescence recovery after photobleaching (FRAP): "**FRAPA-rectangle ROI, single exp**"; "**FRAPA- rectangle ROI, double exp**"; "**FRAPA-oval ROI, single exp**"; "**FRAPA-oval ROI, double exp**" (see Further processing of FRAP data). New models can be added to the program from the "Settings" panel, which is located on the right, next to the "Results" and "Repeats" charts. By clicking on the button, a pop up window "Solver Functions" will appear. The protocol is added by clicking "Save" or "Save As New". Unlimited number of constants and variables can be evaluated during the fitting process. Variable "t" is reserved for the X axis values, and can't be used, all other letters are free for defining new variables. New variable is added by typing the name followed by ";"in the "Variables" text box. If more than one variable is defined, all of them must be separated with ";". Mathematical models (as described below) can be saved in txt. files and quickly loaded and stored in CellTool. This is done by clicking on the "Load" button in the upper left corner of the "Solver Functions" window.

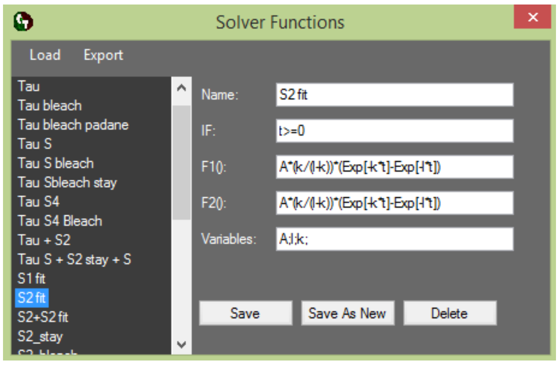

We provide a txt. file (Supplementary file 2.txt) with a number of CRC model equations. The

CRC model describes protein recruitment to damage as a series of up to three consecutive reactions, a period of residence at the lesion, and a reaction through which the protein is removed. Tau_S2, Tau_S3, and Tau_S4 are CRC models that include one, two, and three reactions of recruitment, respectively. Their general equation is shown below.

$$S_l(t,\tau) = A \begin{cases} 1 - \sum_{i=1}^{l-1} \left(\prod_{j=1, j \neq i}^{l-1} \frac{k_j}{(k_j - k_i)} \right) e^{-k_i t} & \text{if } t < \tau, \\ -\sum_{i=1}^{l-1} \left(\prod_{j=1, j \neq i}^{l-1} \frac{k_j}{(k_j - k_i)} \right) e^{-k_i t} & \\ +\sum_{i=1}^{l} \left(\prod_{j=1, j \neq i}^{l} \frac{k_j}{(k_j - k_i)} \right) e^{k_i(\tau - t)} & \text{if } t \geq \tau. \end{cases}$$

We have also included models that describe the behavior of a protein that is recruited to the lesion via several consecutive reactions but does not dissociate from the lesion. These models, termed S_2 to S_6, differ from each other based on the number of recruitment reactions (up to five) and can be described via the following general equation:

$$k_\ell = 0 \rightarrow S_\ell(t) = A\left(1 - \sum_{i=1}^{\ell-1} e^{-k_i t} \prod_{j=1, j \neq i}^{\ell-1} \frac{k_j}{(k_j - k_i)}\right)$$

Our solver supports the following arithmetic operations: addition („+“), subtraction („-“), multiplication („*“), division („/“), exponentiation („Pow[args]“,”Exp[val]”) and square root (“Sqrt[val]”). “If statement” can also be used. The conditions of the “if statement” must be written in the “IF” text box. If the condition is fulfilled the first equation “F1()” will be used for the calculations, otherwise the second equation “F2()” will be used. If this statement is not required, in the “IF” text box only “t>=0” must be written. **Important:** When the first custom mathematical model is ready it can be saved with the “Save” button. For the next model to be saved, it has to be written over the old one and “Save As New” must be pressed afterwards. If “Save As New” is pressed and the name of the new model is the same as the old one both of them will be with the same name. If only “Save” is clicked it will rewrite the new model over the old one.

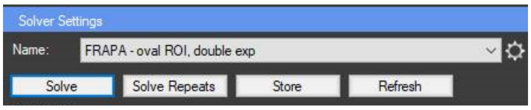

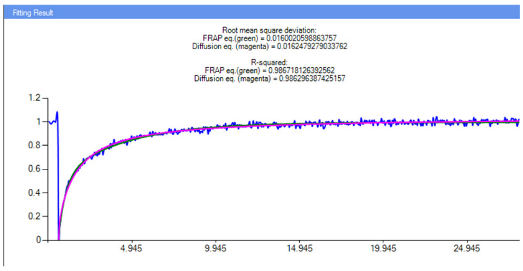

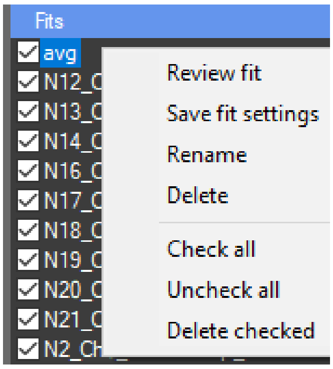

Once created, the mathematical model can be selected from the "Settings" panel. **Important:** Proper initial values to the variables must be assigned in the "Variables" panel. Pressing the button "Solve" will start the solving algorithm. The current average curve (from the "Results" chart) will be used as source data. When the fit data is calculated the result can be seen in the "Fitting Result" chart. The result can be stored in the "Fits" panel (located under the "Settings" panel) by pressing the "Store" button. The button "Solve repeats" activates the solving algorithm that uses the current fit variables and solves the equation for all samples in the "Repeats chart". The fits for all the repeats are automatically stored in the "Fits" panel and can be viewed on the "Fitting result" chart one at a time by double click. Multiple fits can be stored in the "Fits" panel. The fits can also be reviewed with the "Review fit" option in the "Fits" panel context menu. The menu can be activated by right mouse click on a fit in the "Fits" panel and can be used for renaming and deleting stored fits. Also fit settings can be stored in an existing fit by pressing "Save fit settings".

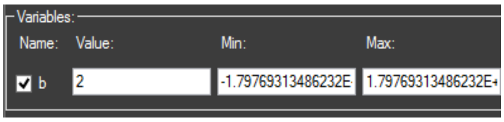

The "Variables" panel provides access to the values of the variables used to calculate the current fit curve. The value of all variables can be manually assigned in order to improve the performance of the solving algorithm. Also the range for variation by the algorithm can be set (the defaults in "Min:" "Max:" are the minimal and the maximal possible values). If the checkbox next to the variable name is unchecked this variable becomes constant and will not be variated by the solving algorithm.

OUTPUT FILE STRUCTURE:

The results from the "Results Extractor" can be exported as TAB-delimited text file and will contain: All samples, the average curve and the standard deviation. For the "Repeats" chart only the results from the visible curves (filtered) and checked files in the "Data" panel will be calculated and exported. Also samples (F10_1; F10_2), average curve (Mean), standard deviation (StDev), normalized samples (nF10_1; nF10_2), normalized average curve (nMean), normalized standard deviation (nnStDev) and double normalized average curve (nnMean) are included in the file.

For every calculated fit another 5 columns (or 6 for FRAP) are included: the variables names (Const_[name of fit]), the variables values (Value_[name of fit]), the X axis data (Xvals_[name of fit]), the Y axis raw data (Raw_[name of fit]), and the calculated Y axis fit data (Fit_[name of fit] or if FRAP model is used there are two columns fit data - Fit_FRAP_eq_[name of fit] and Fit_Diffusion_eq_[name of fit]). Below the constants additional statistical information about the fit is available - root mean square deviation, coefficient of correlation, R – squared (the equations are shown below). For FRAP, because two equations are used for the fitting, all these values are calculated for both of them. If the set is not FRAP set, the maximum Y axis value, the half time of recruitment, the half time of removal and the recalculated time of removal (if the fit curve never reaches the 0 Y axis value) are also calculated. The formulas used in the mathematical model are also shown. Example output file structure:

For FRAPA:

DataTable: T(sec.)/External															
Names:	F10_1.txt_	F10_2.txt_	Mean	StDev	nF10_1.txt	nF10_2.txt	nMean	nStDev	nnMean	Const_fit	Value_fit	Xvals_fit	Raw_fit	Fit_FRAP_eq_fit	Fit_Diffusion_eq_fit
Comments:	gr: 0.7231	gr: 0.7122593911384 |			gr: 0.7231	gr: 0.7122593911384 | bd: 0.824552204236826									
0	1.009171	0.989257	1	0.01	1.009171	0.989257	0.999214	0.014081	0.851687	w	3.44	0	1.0019	0	0
0.13	0.987396	1.020069	1	0.02	0.987396	1.020069	1.003732	0.023103	0.855538	from	5	0.13	0.9826	0	0
0.26	1.088507	0.985445	1.04	0.07	1.088507	0.985445	1.036976	0.072875	0.883873	Io	0.996	0.26	1.0057	0	0
0.39	0.99433	1.009728	1	0.01	0.99433	1.009728	1.002029	0.010888	0.854086	A	0.4633	0.39	0.9993	0	0
0.52	0.919395	0.995776	0.96	0.05	0.919395	0.995776	0.957585	0.05401	0.816204	a	0.2581	0.52	1.0112	0	0
0.575	0	0	0	0	0	0	0	0	0	B	0.501	0.575	0	0	0
0.63	0.220713	0.068237	0.14	0.11	0.220713	0.068237	0.144475	0.107817	0.123144	b	2.3029	0.63	0.0986	0.101411828	0.142976584
0.685	0.21254	0.170947	0.19	0.03	0.21254	0.170947	0.191743	0.029411	0.163434	T1/2	0.6609	0.685	0.1611	0.160126997	0.20142682
0.74	0.279326	0.169767	0.22	0.08	0.279326	0.169767	0.224546	0.07747	0.191394	I	1.0398	0.74	0.2331	0.212529376	0.245738428
0.795	0.234587	0.203443	0.22	0.02	0.234587	0.203443	0.219015	0.022023	0.186679	D	3.2193	0.795	0.2757	0.259360505	0.282633415
0.85	0.350486	0.27436	0.31	0.05	0.350486	0.27436	0.312423	0.053829	0.266296			0.85	0.3148	0.301273839	0.314722829
0.905	0.375757	0.257558	0.32	0.08	0.375757	0.257558	0.316657	0.083579	0.269905	Root mean square deviation:		0.905	0.3406	0.338845225	0.343347888
0.96	0.314538	0.3695	0.34	0.04	0.314538	0.3695	0.342019	0.038864	0.291522	FRAP eq.	0.016	0.96	0.3751	0.372582132	0.369305181
1.015	0.45885	0.467448	0.46	0.01	0.45885	0.467448	0.463149	0.00608	0.394768	Diffusion eq.	0.0162	1.015	0.4174	0.402931785	0.393112538
1.07	0.445889	0.555663	0.5	0.08	0.445889	0.555663	0.500776	0.077622	0.42684			1.07	0.4527	0.43028833	0.415128335
1.125	0.579156	0.480576	0.53	0.07	0.579156	0.480576	0.529866	0.069706	0.451635	Coefficient of correlation:		1.125	0.4668	0.454999147	0.435612719
1.18	0.567536	0.461699	0.51	0.07	0.567536	0.461699	0.514618	0.074838	0.438638	FRAP eq.	0.9933	1.18	0.5021	0.47737041	0.454762037
1.235	0.501146	0.511747	0.51	0.01	0.501146	0.511747	0.506446	0.007496	0.431673	Diffusion eq.	0.9931	1.235	0.4935	0.497671991	0.472729492
1.29	0.614412	0.504266	0.56	0.08	0.614412	0.504266	0.559339	0.077885	0.476757			1.29	0.5075	0.516141775	0.489638148
1.345	0.526351	0.50591	0.52	0.01	0.526351	0.50591	0.51613	0.014454	0.439927	R-squared:		1.345	0.5199	0.532989464	0.505589384
1.4	0.575857	0.698994	0.64	0.09	0.575857	0.698994	0.637425	0.087071	0.543314	FRAP eq.	0.9867	1.4	0.5464	0.548399926	0.520668514
1.455	0.606602	0.584482	0.6	0.02	0.606602	0.584482	0.595542	0.015641	0.507614	Diffusion eq.	0.9863	1.455	0.5503	0.562536153	0.534948639
1.51	0.596371	0.55277	0.57	0.03	0.596371	0.55277	0.57457	0.030831	0.489739			1.51	0.5554	0.575541857	0.548493332
1.565	0.610807	0.722751	0.67	0.08	0.610807	0.722751	0.666779	0.079156	0.568333	FRAP eq.:	f(t)=Io*(1	1.565	0.5666	0.587543765	0.561358554
1.62	0.683854	0.683081	0.68	0	0.683854	0.683081	0.683467	0.000546	0.582558	Diffusion eq.:	f(t)=I*Ex	1.62	0.5895	0.598653636	0.573594056

All other Fits:

DataTable: T (sec.)/Mean														
Names:	Multi:	Multi	Mean	StDev	nMulti	nMulti	nMean	nStDev	nnMean	Const_fit	Value_fit	Xvals_fit	Raw_fit	Fit_fit
Comments:														
0	315	333	321	31.8	0.274	0.27	0.267	0.04	0.2873	A	1087.61	0	320.84	####
1	324	334	325	35.7	0.282	0.27	0.271	0.043	0.2912	k	85.6896	1	325.16	92.5
2	327	348	334	28.4	0.285	0.28	0.278	0.038	0.2992	l	0.08997	2	334.48	178
3	347	334	351	29.9	0.303	0.27	0.292	0.037	0.3138	m	#######	3	351.13	256
4	370	370	379	30.6	0.323	0.3	0.314	0.031	0.3377	n	-1.79769	4	378.66	328
5	424	412	424	42.7	0.37	0.33	0.351	0.035	0.3776			5	424	393
6	447	415	458	64.2	0.39	0.34	0.378	0.042	0.4072	Root mean square deviation	26.7424	6	458.33	453
7	498	444	503	83.8	0.434	0.36	0.415	0.054	0.4462	Coefficient of correlation:	0.98164	7	502.85	508
8	545	499	549	95.2	0.475	0.4	0.453	0.062	0.4873	R - squared:	0.96361	8	549.39	557
9	582	524	596	104	0.508	0.43	0.491	0.066	0.5284			9	596.07	603
10	652	557	638	119	0.569	0.45	0.525	0.075	0.5653	IF:	t>=0	10	638.21	645
11	682	590	672	123	0.595	0.48	0.553	0.076	0.5952	f1:	(A*k*l/((I	11	672.31	683
12	730	599	708	136	0.637	0.49	0.583	0.085	0.6269	f2	(A*k*l/((I	12	708.13	717
13	771	622	734	135	0.673	0.5	0.604	0.084	0.6495			13	733.82	749
14	789	636	763	144	0.688	0.52	0.627	0.089	0.675	Maximum	1082.97	14	762.68	778
15	817	688	799	154	0.712	0.56	0.656	0.094	0.7064	HalfTimeOfRecruitment	8	15	799.05	805
16	832	703	811	151	0.726	0.57	0.666	0.09	0.7166	HalfTimeOfRemoval	NaN	16	810.52	829
17	837	743	829	150	0.73	0.6	0.681	0.088	0.7332	HalfTimeOfRemoval(Recalculated)	NaN	17	829.09	851

STATISTICAL EQUATIONS USED FOR THE CALCULATION OF THE RESULTS:

Standard deviation

$$\sqrt{\frac{\sum_{i=1}^{N}(y_i - \bar{y})^2}{N-1}}$$

Where:

$\bar{y}$ is the sample mean;

y_i is the individual sample point indexed with i;

N is the sample size;

Root mean square deviation

$$\sqrt{\frac{\sum_{i=1}^{N}(\hat{y}_i - y_i)^2}{N}}$$

Where:

$\hat{y}$ are the predicted values;

y_i is the individual sample point indexed with i;

N is the number of observations;

Correlation coefficient

$$\frac{\sum_{i=1}^{N}(x_i - \bar{x})(y_i - \bar{y})}{\sqrt{\sum_{i=1}^{N}(x_i - \bar{x})^2 \sum_{i=1}^{N}(y_i - \bar{y})^2}}$$

Where:

N is the sample size;

x_i and y_i are the individual sample points

indexed with i;
$\bar{x}$ and $\bar{y}$ are the sample means;
N is the number of observations;

R - squared

$$\left(\frac{\sum_{i=1}^{N}(x_i-\bar{x})(y_i-\bar{y})}{\sqrt{\sum_{i=1}^{N}(x_i-\bar{x})^2\sum_{i=1}^{N}(y_i-\bar{y})^2}}\right)^2$$

Where:
N is the sample size;
x_i and y_i are the individual sample points indexed with i;
$\bar{x}$ and $\bar{y}$ are the sample means;
N is the number of observations;

EXAMPLES

MEASURING DNA-REPAIR FOCI

The DNA of every cell is under a constant attack by various mutagenic factors which damage the DNA and can cause cell cycle arrest and even cell death. Accumulation of DNA damage is the basis for cancer development and one of the reasons for aging of the organisms. In order to preserve the integrity of their DNA, cells have evolved an impressive array of DNA repair pathways, which are precisely coordinated with the progression of the cell cycle. Recently published article demonstrates a method for characterization and evaluation of the process of DNA repair by using time-laps fluorescence microscopy of living cells [1]. The model object for these studies is the "HeLa Kyoto" cell line. The cells are transfected with the genes for the proteins of interest fused with a fluorescent protein (EGFP or mCherry). Ultraviolet laser is used to induce lesions in DNA by irradiation (IR) of a small part of the cell nucleus. The expression levels and localization of these proteins are detected due to the fluorescent protein component by measuring the intensity of the pixels in the area of damage. The proteins involved in the process of DNA repair can be characterized by measuring the kinetics of recruitment and removal at the sides of DNA lesions.

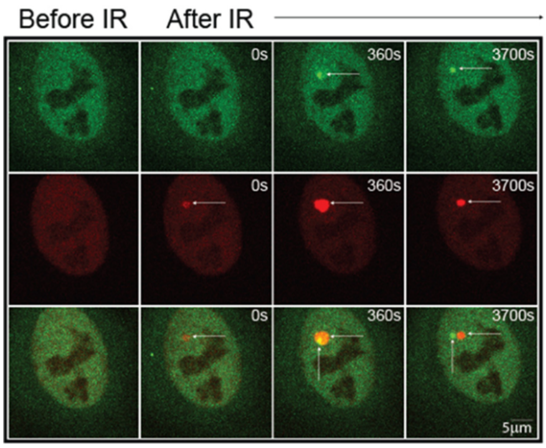

Here we propose the following protocol for semi-automatic analysis of DNA-repair foci.

Properties of the test images: 16-bit grayscale TIF; 3 focal planes with 0.5 um between each; Time interval between 1s and 5s depending on the recruitment time of the examined protein. The images are of living HeLa Kyoto cells acquired with a spinning disk confocal microscope with a UV laser module and recorded with the IQ3 software of Andor. Detailed information for the test images like laser exposure, UV laser intensity, ext. can be found in the image metadata (see Metadata).

Test data sets:

Test data set can be downloaded as an archive from the following URL:

https://dnarepair.bas.bg/software/CellTool/DataSet/MP_DataSet.rar

The **MP_DataSet** archive contains the following test data:

1. **RawDataSet_RNF168** folder – with the raw images from the microscope;
2. **CroppedDataSet_RNF168** folder – with cropped single cell images with Maximum intensity projection;
3. **ProcessedDataSet_RNF168** folder - with the processed images and results text files;
4. **Results_RNF168.CTData** – the input file for the "Results Extractor" with NO calculated fits;
5. **Fit_Results_RNF168.CTData** – the input file for the "Results Extractor" with calculated fits;
6. **Fit_Results_RNF168.txt** - exported text file from the "Result Extractor" containing all the measurements and fits;

IMAGE ANALYSIS:

1. Download the test data archive **MP_DataSet** and extract it to the hard drive.
2. Load the image stack (2_RNF168.tif):
   a. From the "Data sources" panel press "Add work directory" icon and browse to the RawDataSet_RNF168 folder with the sample images on the computer. Select "OK". The chosen folder will now be visible in the "Directory Explorer" of CellTool. Double clicking on the folder will expand it and the images in it will be visible. Double clicking on the 2_RNF168.tif image will open it in the work screen.
   b. The image can also be opened by dragging it from the "File Explorer" of the computer to the work screen.

3. The raw images are in 3 focal planes (Z stack). Maximum intensity projection must be performed for minimizing the effect of eventual vertical movement of the DNA-repair foci.
   - From the Top Menu press “Edit”> “Projection”. Select “Z stack” for data set, and “Maximum” for type of projection.
4. Crop step: There are multiple cells in the original image, the processing can be performed directly on it or the cells can be cropped from the original image, saved as different images and then processed (use an image/s from the **CroppedDataSet_RNF168** folder if you want to skip this step)
   - From the Tool strip menu select “Static ROI” and “Rectangular ROI” to draw the ROI.

     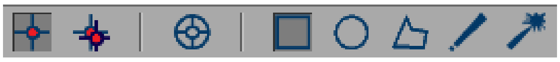

   - All of the cell nuclei that need to be measured have to be added to the “ROI Manager” and selected (see ROI Manager).
   - From the top menu choose “Edit”> “Crop”. All of the selected ROIs will be exported as new images in new tabs.
   - Save every cell as a different image “File”> “Save” or “Save as” if you want to change the image name (we recommend “Save as” if you are working in the original image, always keep the original image intact).
   - Choose one of the cells for further processing.
5. Image filters need to be applied in order to minimize the effect of the noise over the further image processing.
   - Press the icon for the “Processed image” 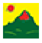 from the tab page menu to make it visible and select the processed image to enable the options in the “Properties” panel.
   - We recommend Gaussian blur 5x5. In the “Properties” panel in “Filters”> “Convolutions” select “Smooth”> “Gaussian blur 5x5”.
6. Image segmentation is performed by using K-means or Otsu thresholding with one threshold. As a result, the cell nuclei must be successfully segmented.
   - In the “Segmentation” panel for “Method” choose “Otsu thresholding” or “K-means”, then an “Options:” panel will be enabled in it set the number of “Thresholds” to 1.

     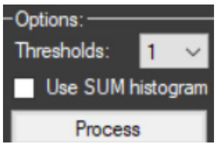

   - “Use SUM histogram” must be unchecked, for the segmentation only the current frame will be used. The image should be on the first frame, because there is no DNA repair focus there.
   - Press “Process” to start the segmentation. If the segmentation is not satisfying the threshold in the “Thresholds” panel can be manually adjusted.

     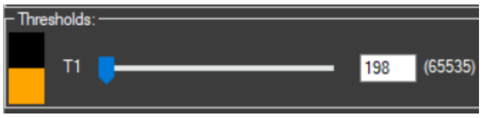

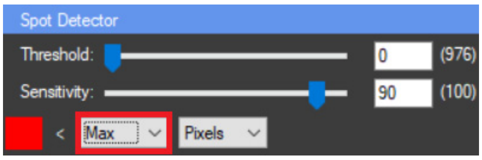

7. From the "Spot Detector", the "Threshold" and "Sensitivity" can be modified for better detection. Move the track bar of the image forward a few frames (10-20) to a point where the DNA repair focus in the cell nucleus is visible to make sure it's segmented.
   - The combo box under "Sensitivity" must be set to "Max". The highest intensity value that occurs in the image. This is the starting point for the algorithm.
   - The "Threshold" can be 0 for this set of images. It depends on the intensity of the signal.
   - The "Sensitivity" must be set to 90 for smoothing of the histogram and better results.
8. Tracking step: The automatic algorithm will track the DNA-repair focus throughout the image stack.
   - From the Tool strip menu select "Tracking ROI" and "Oval ROI", enable the "Stack ROI" option to draw the ROIs (see ROI Manager).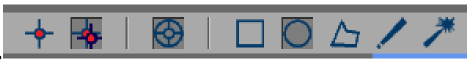
   - With right mouse click in the segmented image (make sure it's active) select the DNA-repair focus (it will be colored in red).
   - See if the algorithm tracked the DNA-repair focus correctly. If not, adjust the tracking settings.
   - Add the ROI to the ROI manager (Ctrl + T).
9. Results calculation: Here we are using a predefined formula to eliminate some of the postprocessing steps:
   - Enable the "Results chart" from the tab page menu to view the result.
   - Select the chart to enable the "Chart Properties" panel.
   - Open the "Function editor" by pressing from the "Chart Properties" panel (see Results Chart Settings).
   - In the f(x) text box add the following formula:

     ((Mean[][0][]-Mean[][1][])-(Mean[][0][N]-Mean[][1][N]))*Area[][0][]

     (The N must be replaced with 5. N is the numeric value indicating the frame of laser microirradiation. For the test data the microirradiation is at the end of frame 5. That is why the bleach effect is visible on frame 6.)
   - Give the function the name "Micropoint" and press "Add new function".
   - Select it from the Y axis combo box in the "Chart Properties" panel.
   - Select T(sec) from the X axis combo box. If the time interval is not correct, it can be changed from the metadata.

Important: After this step, the settings can be stored as new protocol and can be applied to the other images automatically (see Auto Processing).

10. Export all the results as Text files by pressing “File”> “Auto Export” (see File Menu).
11. Save the image with the changes. “File”> “Save” or “Save as”.

Important Note: Saving the image in CellTool will keep all the settings for that image like ROIs, Segmentation, Results chart properties, everything. And the user can always return later to view the image or extract more results if needed. There are preprocessed images in the **ProcessedDataSet_RNF168** folder.

FURTHER PROCESSING OF THE ACQUIRED DATA IN THE RESULTS EXTRACTOR TAB.

The Results Extractor Tab can be used to extract the information from the results files and to calculate the mean value of each repeat of the experiment (see Output file structure). The raw data can be fitted to a mathematical model (see Solver). For the test data CRC modeling of protein kinetics was used, as described in the Aleksandrov’s paper [16]. The formula can be loaded automatically by opening the “**Fit_Results_RNF168.CTData**” file from the processed data set or manually in the “**Results_RNF168.CTData**”. The structure of the model is as follows:

IF: t>=0
F1(): (A*k*l*m)*((1/((l-k)*(m-k)*(n-k)))*Exp[-k*t]+(1/((k-l)*(m-l)*(n-l)))*Exp[-l*t]+(1/((k-m)*(l-m)*(n-m)))*Exp[-m*t]+(1/((k-n)*(l-n)*(m-n)))*Exp[-n*t])
F2():0
Variables: A;k;l;m;n;

Processing of the results and fitting them to a custom mathematical model in the “Results Extractor”:

1. Open the “Results Extractor”: “File”> “New Results Extractor”.
2. Load the results to the “Results Extractor”:
   a. From the ProcessedDataSet, the files can be automatically loaded:
   - Press “Add work directory” and browse to the folder with the text files.
   b. Test results files can also be used: **Results_RNF168.CTData** with the raw results or **Fit_Results_RNF168.CTData** with a preloaded formula and calculated fits.
3. For the tutorial we are using the test results file: **Results_RNF168.CTData** with no preloaded formula.
   - Press “Open”, browse to the “.CTData” file and select it or just drag and drop the file in the program.
4. Remove unwanted charts from the “Data” panel by unchecking them. They will not be included in the exported file.
5. From the “Settings” panel “Normalization” must be checked, “Max=1 & Mean=0” and “Double Normalize” are optional.

6. Check “Subset” in the “Settings” panel and in the pop up window for “Start from:” type 25 sec and for “End at:” 2000 sec. We are doing that because the micropoint irradiation is on the 5th frame which is the 25th second and 2000sec because the length of the two raw tiff files the cells are generated from is different and it need to be the same.
7. Fitting of the result (Optional):
   - From the “Solver Settings” panel press the 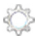 next to the fit name combo box to load the formula:
     Name: Fit1
     IF: t>=0
     F1(): (A*k*l*m)*((1/((l-k)*(m-k)*(n-k)))*Exp[-k*t]+(1/((k-l)*(m-l)*(n-l)))*Exp[-l*t]+(1/((k-m)*(l-m)*(n-m)))*Exp[-m*t]+(1/((k-n)*(l-n)*(m-n)))*Exp[-n*t])
     F2():0
     Variables: A;k;l;m;n;
   - Type random different values in the “Variables:” panel (exp. A:1, k:0.5, l:0.6, m:0.7, n:0) and press “Solve”.
   - The fit will appear on the “Fitting Result” chart.
   - Press “Store” and the new fit will appear in the “Fits” panel.
   - Press “Solve Repeats” to fit the individual repeats. It will use the current values to find the closest ones for every individual repeat. They will automatically appear in the “Fits” panel with the names of the repeats.
   - With double click on the individual repeats from the “Fits” panel they can be viewed one by one in the “Fitting Result” chart.
8. Saving the results:
   - With the button “Save” the measures made on the **Results_RNF168.CTData** can be saved as new file, that includes the fits and the formula used for them and all other changes made from the user.
   - With the “Export” button all the information in the CTData file will be exported as a tab-delimited text file that includes the fits, Normalization, Standard deviation, ext.
9. There is also a previously generated text file containing that information: Fit_Results_RNF168.txt (see Output File Structure).

FRAP ANALYSIS

Fluorescence recovery after photobleaching (FRAP) is a method for determining the kinetics of diffusion through tissues or cells. It is capable of quantifying the two dimensional lateral diffusions of a molecule thin film containing fluorescently labeled probes, or to examine single cells. This technique is very useful in biological studies of cell membrane diffusion and protein binding. Here we propose a protocol for semi-automatic image analysis of FRAP of nuclear proteins fused with fluorescent protein.

Properties of the test images: 16-bit grayscale TIF; 1 focal plane. The images are of living HeLa Kyoto cells acquired with a spinning disk confocal microscope and recorded with the IQ3 software of Andor. Detailed information for the test images like laser exposure for instance can be found in the image metadata (see Metadata).

Test data sets:

Test data set can be downloaded as an archive from the following URL:

https://dnarepair.bas.bg/software/CellTool/DataSet/FRAP_DataSet.rar

The **FRAP_DataSet** archive contains the following test data:

1. **RawDataSet_FRAP** folder – with one raw test image from the microscope;
2. **CroppedDataSet_FRAP** folder – with cropped single cell images;
3. **ProcessedDataSet_FRAP** folder - with the processed images and results text files;
4. **Results_FRAP.CTData** – the input file for the "Results Extractor" with NO calculated fits;
5. **Fit_Results_FRAP.CTData** – the input file for the "Results Extractor" with calculated fits;
6. **Fit_Results_ FRAP.txt** - exported text file from the "Result Extractor" containing all the measurements and fits;

The **RawDataSet_FRAP_All** archive contains all the raw images used for creating the CroppedDataSet can be downloaded from the following URL:

https://dnarepair.bas.bg/software/CellTool/DataSet/RawDataSet_FRAP_All.rar

IMAGE ANALYSIS:

1. Download the test data archive **FRAP_DataSet** and extract it to the hard drive.
2. Load the image stack (FRAP.tif):

a. From the “Data sources” panel press “Add work directory” icon and browse to the RawDataSet_FRAP folder with the sample image on the computer. Select “OK”. The chosen folder will now be visible in the “Directory Explorer” of CellTool. Double clicking on the folder will expand it and the image in it will be visible. Double clicking on “FRAP.tif” image will open it in the work screen.
b. The image can also be opened by dragging it from the “File Explorer” of the computer to the work screen.

3. Crop step: There is one cell in the original image that is bleached, the processing can be performed directly on it or the cell can be cropped from the original image, saved as different image and then processed (use an image/s from the **CroppedDataSet_FRAP** folder if you want to skip this step. Load it the same way as the FRAP.tif).
   - From the Tool strip menu select “Static ROI” and “Rectangular ROI” to draw the ROI.

   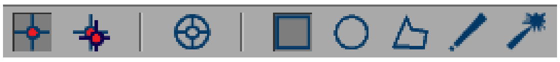

   - The cell nucleus that needs to be measured has to be added to the “ROI Manager” and selected (see ROI Manager).
   - From the top menu choose “Edit”> “Crop”. The selected ROI will be exported as a new image in a new tab.
   - Save the cell as a different image “File”> “Save” or “Save as” if you want to change the image name (we recommend “Save as” if you are working in the original image, always keep the original image intact).
4. Image filters need to be applied in order to minimize the effect of the noise over the further image processing.
   - Press the icon for the “Processed image” 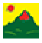 from the tab page menu to make it visible and select the processed image to enable the options in the “Properties” panel.
   - We recommend Gaussian blur 5x5. In the Properties panel in “Filters”> “Convolutions” select “Smooth”> “Gaussian blur 5x5”.
5. Image segmentation is performed by using K-means or Otsu thresholding with one threshold. As a result, the cell nucleus must be successfully segmented.

   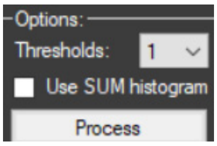

   - In the “Segmentation” panel for “Method” choose “Otsu thresholding” or “K-means”, then an “Options:” panel will be enabled in it set the number of “Thresholds” to 1.
   - “Use SUM histogram” can be checked, for the segmentation all frames will be calculated.
   - Press “Process” to start the segmentation. If the segmentation is not satisfying the threshold in the “Thresholds” panel can be manually adjusted.

   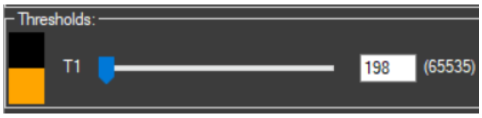

6. Measuring the intensity change at the region of photobleaching.

- In the Tool strip menu select “Static ROI” and “Rectangular ROI” (see ROI Manager).

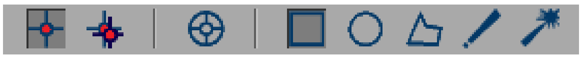

- Hold the right mouse button in the raw image (make sure it’s active) and draw a ROI at the region of photobleaching. The “Auto find” option in the context menu of the ROI manager can be used for convenience.
- Add the ROI to the ROI manager (Ctrl + T).

7. Enable the “Results chart”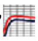 from the tab page menu to view the result.
8. Measuring the intensity of the whole cell nucleus. The automatic algorithm will track the segmented nucleus throughout the image stack.
   - In the Tool strip menu select “Tracking ROI” and “Magic wand ROI” (see ROI Manager).

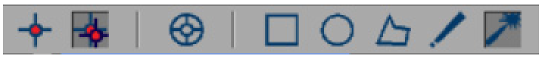

   - With right mouse click in the segmented image (make sure it’s active) select the nucleus (it will be colored in yellow).
   - See if the algorithm tracked the nucleus correctly. If not, adjust the tracking settings.
   - Add the ROI to the ROI manager (Ctrl + T).
9. Measuring the intensity of the background noise.
   - In the Tool strip menu select “Static ROI” and “Rectangular ROI” (see ROI Manager).

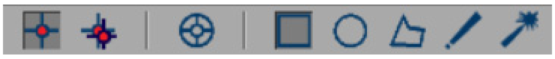

   - Draw the ROI somewhere in the background.
   - Add the ROI to the ROI manager (Ctrl + T).
10. Select the Results chart to enable its “Properties”. Select “T(sec)” from the X axis combo box and “Mean” from Y axis combo box. If the time interval is not correct, you can change it from the metadata.

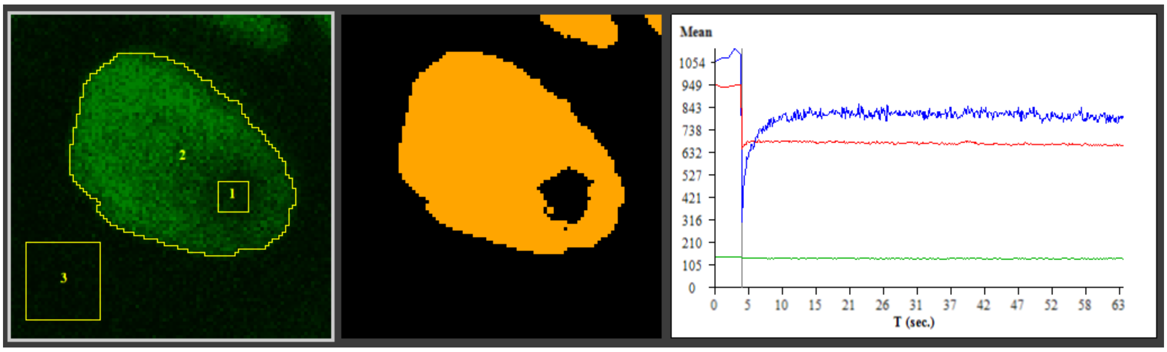

Important: after this step the settings can be stored as new protocol and can be applied to the other images automatically (see auto processing).

11. Export all the results as Text files by pressing “File”> “Auto Export” (see File Menu).

12. Save the image with the changes. “File”> “Save” or “Save as”. (We recommend not to save over the original image files).

FURTHER PROCESSING OF THE FRAPA DATA IN THE RESULTS EXTRACTOR:

The Results Extractor Tab can be used to extract the information from the text files, perform normalization, calculate the mean value of each repeat of the experiment and fit the data to a FRAP model.

1. Open the “Results Extractor”: “File”> “New Results Extractor”.
2. Load the results to the “Results Extractor”:
   - A test results file can be used: **Fit_Results_FRAP.CTData** with calculated fits or **Results_FRAP.CTData** with the raw results.
   - Press the button “Open” in the Results extractor and browse to the **Results_FRAP.CTData** file and select it.
3. In the “Settings” panel check “Subset” if only a portion of the result displayed on the charts is needed. Nothing else needs to be specified.
4. Performing “Acquisition bleaching correction” in order to normalize the data by applying two formulas, one for **Double Normalization** and one for **Full Scale Normalization** [2]:

$$I_{norm}^{double}(t) = \frac{Iref\_pre}{Iref(t) - Iback(t)} \cdot \frac{Ifrap(t) - Iback(t)}{Ifrap\_pre}$$

Where:

$I_{norm}^{double}(t)$ – **double normalized** intensity;

$Ifrap(t)$ – measured average intensity inside the bleached spot;

$Iref(t)$ – measured average reference or whole studied compartment (cell, nucleus, etc.) intensity;

$Iback(t)$ – measured average background intensity outside the cell;

Subscript *_pre* means the averaging of intensity in the corresponding ROI before bleach moment after subtraction of background intensity;

$$I_{norm}^{full\ scale}(t) = \frac{I_{norm}^{double}(t) - I_{norm}^{double}(t_{bleach})}{1 - I_{norm}^{double}(t_{bleach})}$$

Where:

$I_{norm}^{full\ scale}(t)$ - **full scale normalized** intensity;

$I_{norm}^{double}(t)$ - double normalized intensity;

$I_{norm}^{double}(t_{bleach})$ - double normalized intensity at the time of the bleach;

- Right click in the “Data” panel of the Results Extractor to open a Context Menu
- In it choose “Acquisition bleaching correction”

5. Three parameters need to be predefined in the "Acquisition bleaching correction" window:
   - Next to "Bleaching at:", the number of the frame of the bleaching must be specified. In our case that frame is 5.
   - Next to "Pre-Bleach:" the amount of frames before bleaching must be specified for the pre bleach averaging. There must be at least one frame before the bleaching, any number from 1 to 5 included will suffice in our case.
   - Next to "Post-Bleach:" the amount of frames after bleaching must be specified for the post bleach averaging.

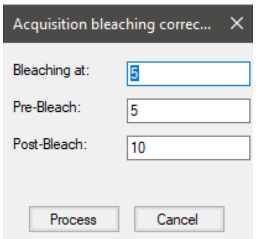

Important: The **gap ratio** and the **bleaching depth** are automatically calculated and stored as comment for each data series, they will be exported in the text file also. They are calculated with the following formulas [3]:

$$bd = \frac{Ifrap\_pre - Ifrap(t_{bleach})}{Ifrap\_pre}$$

Where:

bd – **bleaching depth**;

$Ifrap\_pre$ - averaging of the measured average intensity inside the bleached spot before bleach moment after subtraction of background intensity;

$Ifrap(t_{bleach})$ – measured average intensity inside the bleached spot at the time of the bleach after subtraction of background intensity;

$$gr = \frac{Ifrap\_post}{Ifrap\_pre}$$

Where:

gr – **gap ratio**;

$Ifrap\_pre$ - averaging of the reference or whole studied compartment (cell, nucleus, etc.) intensity before bleach moment after subtraction of background intensity;

$Ifrap\_post$ - averaging of the reference or whole studied compartment (cell, nucleus, etc.) intensity after bleach moment after subtraction of background intensity;

6. Remove unwanted curves from the "Data" panel by unchecking them. They will not be included in the exported file.
7. Fitting of the result (Optional):
   - Using the predefined models from the "Solver Setting" panel choose the name of the proper model for the fitting.
   - There are four predefined models: "**FRAPA- rectangle ROI, single exp**" ; "**FRAPA- rectangle ROI, double exp**"; "**FRAPA- oval ROI, single exp**"; "**FRAPA- oval ROI, double exp**", that are combination of the following formulas:

One binding state model (single exp) [44, 46]:

$$FRAP(t) = Io(1 - e^{-at})$$

Where:

Io – mobile fraction;

a - unbinding rate constant [s^{-1}];

t – time after the bleaching [s];

Two binding states model (double exp) [44, 46]:

$$FRAP(t) = Io(1 - Ae^{-at} - Be^{-bt})$$

Where:

Io – mobile fraction;

a, b - unbinding rate constants for the two binding states [s^{-1}];

A, B - fractions of bound molecules for the two binding states;

t – time after the bleaching [s];

Diffusion model for rectangular ROI [44, 47]:

$$FRAP(t) = I\left(1 - \sqrt{\frac{w^2}{w^2 + 4\pi Dt}}\right)$$

Where:

I – normalizing coefficient to account the incomplete recovery;

D - diffusion coefficient [$\mu m^2 s^{-1}$];

w - the width of bleach spot [µm];

t – time after the bleaching [s];

Diffusion model for oval ROI [42, 44]:

$$FRAP(t) = I \cdot e^{\frac{w^2}{2Dt}}\left(I_0\left(e^{\frac{w^2}{2Dt}}\right) + I_1\left(e^{\frac{w^2}{2Dt}}\right)\right)$$

Where:

$I_0(x), I_1(x),$ - modified Bessel functions.

I – normalizing coefficient to account the incomplete recovery;

D - diffusion coefficient [$\mu m^2 s^{-1}$];

w - the radius of bleach spot [µm];

t – time after the bleaching [s];

8. In our case we are using the "**FRAPA- rectangle ROI, double exp**" model, because we have two binding states and an oval ROI for the FRAPA region.
9. In the "Variables:" panel, for "w" fill the parameter in **µm** with the correct width for a rectangular ROI or radius for an oval ROI.
   - In our case in "w" we fill the width for a rectangular ROI, which is 2,3 **µm**.
   - In "from" fill the bleaching frame number, in our case, that number is 5.
10. Press "Solve" and the calculated fit will appear on the "Fitting Result" chart.
11. Press "Store" and the new fit will appear in the "Fits" panel.
12. The model can be solved for each repeat individually if the button "Solve repeats" is activated. They will automatically appear in the "Fits" panel with the names of the repeats.
    - Select them one by one from the "Fits" panel to view them on the "Fitting Result" chart.

10. Saving the results:
    - With the button "Save" the measures made in the Results_FRAP.CTData can be saved as new file, that includes the fits and the formula used for them and all other changes made from the user.
    - With the "Export" button all the information from the CTData file will be exported as a tab-delimited text file.
11. There is also a previously generated tab delimited text file containing that information: Fit_Results_FRAP.txt (see Output File Structure).

DEVELOPER GUIDE

Install your favorite IDK for .NetFramework programming. Choose the program language and create a new Class Library Project. Edit the name of assembly from the Project properties by adding *".CTPlugIn"*. CellTool will recognize as plugins only assemblies with suffix *".CTPlugIn.dll"*. Add a reference to CellToolDK.dll (Source code is available in GitHub).

The main class of the plugin must have the following construction (C# example):

```
using CellToolDK;
public class Main
{
    private Transmiter t;
    private TifFileInfo fi;
    private void ApplyChanges()
    {
        //Apply changes and reload image
        t.ReloadImage();
    }
    public void Input(TifFileInfo fi, Transmiter t)
    {
        this.t = t;
        this.fi = fi;
        //Main entrance
        //You can add your code here
    }
}
```

Public void "Input" is the main entrance of the program. This is the void that will start when the plugin is activated from CellTool menu Plugins and here you must add your code. This void has two arguments - TifFileInfo fi and Transmiter t. TifFileInfo fi contains the data from the active image. The transmitter t is used to send back information to CellTool. The command "t.ReloadImage()" can be used to send the modified version of the image back to CellTool.

To install the plugin, start the "CellTool.exe" as administrator and press install button in the PlugIns menu. Browse to your assembly and press the "OK" button.

Example plugin is avaliable here.

TIFFILEINFO CLASS

Name:	Type:	Description:
roiList	List<ROI>[]	Array that contains one List<ROI> for each channel color
ROICounter	int	Available ROI index. When ROI is added this index must be assigned as ID to it and the value of the variable must be increased by 1
frame	int	Current position in the T stack
zValue	int	Current position in the Z stack
cValue	int	Current position in the C stack
loaded	bool	Returns true if the image loading process is finished
available	bool	Returns true if the image is processed at that time
image8bit	byte[][][]	The image is loaded here if the pixel format is 8 bit - [image index][y][x]*
image8bitFilter	byte[][][]	The processed image is loaded here if the pixel format is 8 bit - [image index][y][x]*
image16bit	ushort[][][]	The image is loaded here if the pixel format is 16 bit - [image index][y][x]*
image16bitFilter	ushort[][][]	The processed image is loaded here if the pixel format is 16 bit - [image index][y][x]*
Dir	string	Directory of the image
imageCount	int	The total number of images in the stack
sizeX	int	X size of the image
sizeY	int	Y size of the image
sizeZ	int	Z size of the image stack
umZ	double	Distance between Z slices in um
umXY	double	Size of one pixel in um
sizeC	int	Number of different color stacks in the image
sizeT	int	Size of the T stack
bitsPerPixel	int	Bits per pixel - value can be 8 or 16
LutList	List<Color>	list with the colors of the different color image stack
FileDescription	string	Description tiff tag (code 270) as a string

*Image stack dimension order is XYCZT

ROI CLASS

Name:	Type:	Description:
getID	int	ROI identification number
type	int	Type of the ROI (0 - static and 1 - tracking)
Shape	int	Shape of the ROI (0 - rectangle, 1 - oval, 2 - polygon, 3 - freehand, 4 - magic wand)
Width	int	Width for a rectangular and oval ROI
Height	int	Height for a rectangular and oval ROI
Stack	int	Number of ROI layers
D	int	Distance between the ROI layers in pixels
FromT	int	The start position of the ROI in the Time stack
ToT	int	The end position of the ROI in the Time stack
FromZ	int	The start position of the ROI in the Z stack
ToZ	int	The end position of the ROI in the Z stack
GetLocation(int ImageN)	Point[]	Returns an array with one point (upper left angle of oval and rectangular ROI) or an array with points for every angle of a polygonal ROI for the selected image in the image stack
GetLocationAll()	Point[][]	Returns an array with one point (upper left angle of oval and rectangular ROI) or an array with points for every angle of a polygonal ROI for each image in the image stack

REFERENCES:

16. Aleksandrov, R., et al., *Protein Dynamics in Complex DNA Lesions.* Mol Cell, 2018. **69**(6): p. 1046-1061 e5.

42. Soumpasis, D.M., Theoretical analysis of fluorescence photobleaching recovery experiments. Biophys J, 1983. 41(1): p. 95-7.

44. Halavatyi, A.; Medves, S.; Hoffmann, S.; Apanasovich, V.; Yatskou, M.; Friederich, E. Mathematical model and software FRAPAnalyser for analysis of actin-cytoskeleton dynamics with FRAP experiments. In Proceedings of FEBS/ECF Workshop Mechanics and Dynamics of the Cytoskeleton, Potsdam, Germany, 22–26 June 2008; Potsdam50.

45. Rapsomaniki, M.A., et al., easyFRAP: an interactive, easy-to-use tool for qualitative and quantitative analysis of FRAP data. Bioinformatics, 2012. 28(13): p. 1800-1.

46.. Sprague, B.L., et al., Analysis of binding reactions by fluorescence recovery after photobleaching. Biophys J, 2004. 86(6): p. 3473-95.

47. Ellenberg, J., et al., Nuclear membrane dynamics and reassembly in living cells: targeting of an inner nuclear membrane protein in interphase and mitosis. J Cell Biol, 1997. 138(6): p. 1193-206.
